# Regulation of the Hippo/YAP axis by CXCR7 in the tumorigenesis of gastric cancer

**DOI:** 10.1186/s13046-023-02870-3

**Published:** 2023-11-10

**Authors:** Tianshi Wang, Dehai Wang, Yanan Sun, Ting Zhuang, Xin Li, Huijie Yang, Yifeng Zang, Ziping Liu, Penghe Yang, Chenmiao Zhang, Jiayao Cui, Mingxi Fu, Shuqing Zhang, Peng Su, Zhongbo Li, Jian Zhu, Yinlu Ding

**Affiliations:** 1 Department of General Surgery, The Second Hospital of Shandong University, Cheeloo College of Medicine, Shandong University, Jinan, 250033 China; 2 Xinxiang Key Laboratory of Tumor Migration and Invasion Precision Medicine, School of Medical Technology, Xinxiang Medical University, Xinxiang, 453003 Henan Province P. R. China; 3 Department of Thoracic Surgery, The Second Hospital, Cheeloo College of Medicine, Shandong University, Shandong Province, P. R. China; 4 Department of Pathology, Qilu Hospital, Cheeloo College of Medicine, Shandong University, Jinan, 250033 China; 5 Department of General Surgery, Shengjing Hospital of China Medical University, Shenyang, China

**Keywords:** Hippo signalling, CXCR7, Gastric cancer, LATS, Tumorigenesis

## Abstract

**Background:**

The Hippo pathway is crucial in organ size control and tumorigenesis. Dysregulation of the Hippo/YAP axis is commonly observed in gastric cancer, while effective therapeutic targets for the Hippo/YAP axis are lacking. Identification of reliable drug targets and the underlying mechanisms that could inhibit the activity of the Hippo/YAP axis and gastric cancer progression is urgently needed.

**Methods:**

We used several gastric cancer cell lines and xenograft models and performed immunoblotting, qPCR, and in vivo studies to investigate the function of CXCR7 in gastric cancer progression.

**Results:**

In our current study, we demonstrate that the membrane receptor CXCR7 (C-X-C chemokine receptor 7) is an important modulator of the Hippo/YAP axis. The activation of CXCR7 could stimulate gastric cancer cell progression through the Hippo/YAP axis in vitro and in vivo, while pharmaceutical inhibition of CXCR7 via ACT-1004–1239 could block tumorigenesis in gastric cancer. Molecular studies revealed that the activation of CXCR7 could dephosphorylate YAP and facilitate YAP nuclear accumulation and transcriptional activation in gastric cancer. CXCR7 functions via G-protein Gα_q/11_ and Rho GTPase to activate YAP activity. Interestingly, ChIP assays showed that YAP could bind to the promoter region of CXCR7 and facilitate its gene transcription, which indicates that CXCR7 is both the upstream signalling and downstream target of the Hippo/YAP axis in gastric cancer.

**Conclusion:**

In general, we identified a novel positive feedback loop between CXCR7 and the Hippo/YAP axis, and blockade of CXCR7 could be a plausible strategy for gastric cancer.

**Supplementary Information:**

The online version contains supplementary material available at 10.1186/s13046-023-02870-3.

## Highlight

1: Our study reveals a novel feedback loop between the Hippo pathway and CXCR7 in coordinating tumorigenesis in gastric cancer.

2: Our study reveals that blockade of CXCR7, which impedes membrane signal transduction, could be a promising strategy for gastric cancer therapeutics.

3: Our model that YAP facilitates GPCR transcription, while GPCR in turn facilitates the Hippo/YAP axis, is likely to be a general phenomenon in the cancer research field, which could reveal a broad phenomenon between Hippo signalling and GPCR.

**Supplementary Information:**

The online version contains supplementary material available at 10.1186/s13046-023-02870-3.

## Background

The Hippo pathway is an evolutionarily conserved pathway that was originally identified in Drosophila [1, 2]. Hippo signalling participates in several physiological processes, including cell proliferation, tissue regeneration and organ size control in mammals [3–5]. The key components of Hippo signalling include a series of phospho-kinases, such as MST1/2, LATS1/2 and NF2. When Hippo signalling is activated, the upstream kinases MST1/2 phosphorylate LATS1/2, which subsequently phosphorylates YAP at several serine/threonine sites and facilitates YAP cytosol retention. The phosphorylated YAP protein could associate with β-TrCP for protein degradation. When the Hippo pathway is inhibited, the unphosphorylated YAP protein can shuttle into the nucleus and associate with a few transcription factors, such as TEADs, which induce target gene expression and tumorigenesis [6–9].

Gastric cancer is one of the most frequently diagnosed malignancies worldwide, and 60% of newly diagnosed cases are in China [10–12]. Although this disease can be treated via surgery, the 5-year survival is approximately 30%, making it an urgent issue in cancer research. Although genome-wide association studies and molecular biology studies aim to elucidate the driver molecules in gastric cancer progression, the detailed mechanisms remain to be identified. Dysfunction of the Hippo pathway was commonly observed in human malignancies, including gastric cancer [13–15]. For example, elevated YAP protein levels were found in gastric cancers, which also correlated with lymph node metastasis. The survival data analysis showed that YAP expression correlated with poor survival in gastric cancer. Several biological studies have indicated that YAP is required for the tumorigenic process of gastric cancer, while depletion of YAP expression significantly inhibits cell growth and invasion in gastric cancer [16–18]. Importantly, recent studies have indicated that the Hippo/YAP axis also participates in the transformation process from Helicobacter pylori-induced gastritis to gastric cancer via cytotoxin-associated gene A (Cag A) [19, 20]. Based on the importance of Hippo signalling in gastric cancer, we propose that targeting the Hippo/YAP axis is a promising strategy in the clinic.

Recent studies have focused on developing effective inhibitors of the Hippo/YAP axis in Hippo-driven cancers. According to previous studies, verteporfin has emerged as a promising candidate known for its ability to disrupt YAP-TEAD interactions and modulate target gene expression [21, 22]. Worth noting, verteporfin has already found clinical utility, notably as Visudyne, in the treatment of eye diseases [23, 24]. However, it's crucial to address that despite its clinical use, challenges like photosensitivity and the generation of oxygen species have hindered its broader translation into clinical applications [25, 26]. In addition, VGLL4-like super-TDU was shown to compete with YAP for TEAD binding, while cell membrane penetration and peptide stability in vivo were considered to be potential limits for its clinical applications [27, 28]. Thus, there is still a lack of effective target drugs for the Hippo/YAP axis in human cancer. Based on this, we shifted our strategy to inhibiting the upstream membrane receptors of the Hippo/YAP axis in gastric cancer. In the current study, we identified a novel G-coupled protein receptor member, CXCR7 [29, 30], which promoted gastric cancer progression by activating the YAP axis through the Gα_q/11_-ROCK-LATS cascade. In turn, YAP regulated CXCR7 transcription by binding to its promoter region. Our study elucidated a positive regulatory loop between CXCR7 and YAP to coordinate Hippo signalling activity and tumorigenesis. Importantly, pharmaceutical targeting of CXCR7 could block this feedback loop and inhibit tumorigenesis in gastric cancer. In general, we identified a novel positive feedback loop between CXCR7 and the Hippo/YAP axis, and blockade of CXCR7 could be a plausible strategy for gastric cancer.

## Materials and methods

### Cell culture

MGC803, Hs746T, and HEK-293 T cells were obtained from the American Type Culture Collection (ATCC). MGC803, Hs746T and HEK-293 T cells were cultured in Dulbecco’s modified Eagle’s medium (Thermo Fisher Scientific, Cat: 11,965,092) containing 4.5 g/L glucose and 4 mM L-glutamine (DMEM, 41,965, Life Technologies) and supplemented with 10% foetal bovine serum (FBS, Biological Industries, BISH0744). Cultures were maintained in a humidified incubator at 37 °C with 5% CO_2_. The Power Plex 21 system applied short tandem repeat (STR) analysis to identify all cell lines described in the article. The STR data of MGC803, Hs746T and HEK-293 T cell lines were examined and matched with the STR data of ATCC.

### Cell cryopreservation

MGC803, Hs746T and HEK-293 T cells in the logarithmic growth phase were detached using trypsin–EDTA (Thermo Fisher Scientific, Cat:25,200,072) and suspended in serum-free cell cryopreservation solution (CELLSAVING, cat: C40100). Cell suspensions were aliquoted into cryovials and stored in liquid nitrogen.

### Cell maintenance and passage

Cells were passaged using a standard trypsin–EDTA dissociation protocol. Upon reaching 70–80% confluence, the cells were harvested, washed with prewarmed PBS, and resuspended in fresh culture medium. Subcultures were initiated at a seeding density of 1 × 10^5^ cells/mL.

### RNA extraction and qPCR analysis

Total cellular RNA was extracted using the RNeasy Plus Mini Kit (Tiangen, DP451) according to the specifications provided by the manufacturer. The total RNA obtained in the previous step was reverse transcribed using HiScript II Q RT SuperMix (Vazyme, R223-01). qRT‒PCR was performed using SYBR qPCR Master Mix (Vazyme, Q511-02) under a 7500 Fast Real-Time PCR System (Applied Biosystems, Singapore) machine, and 36B4 was used as an internal reference. The primer sequences designed for the qPCR assay are shown below: 36B4 F: GCA GCA TCT ACA ACC CTG AAG; R: CAC TGG CAA CAT TGC GGA C. CTGF F: ACC GAC TGG AAG ACA CGT TTG; R: CCA GGT CAG CTT CGC AAG G. CYR61 F: GGT CAA AGT TAC CGG GCA GT; R: GGA GGC ATC GAA TCC CAG C. CXCR7 F: CTA TGA CAC GCA CTG CTA CAT C; R: CTG CAC GAG ACT GAC CAC C. ANKRD1 F: CGC ATT TCG GCA AGT TGG AG; R: TCC TGC TTG AAT CAG CCA GAC; YAP F: CAA GAA AGC AGG CTC ACA GAA; R: GCT GGG TGT TAG GGC TTC G. The specifics of all primer pairs were assessed by melting curve analysis.

### Plasmids and siRNA

The CXCR7 and LATS1 plasmids required for the experiments were obtained from HANBIO (https://www.hanbio.net). Plasmids were transiently transfected with Lipofectamine 2000 (1662298, Invitrogen). Specific target genes were thereby knocked down using small interfering RNA. The sequences of CXCR7 siRNA are as follows: (1) CGC ACU GCU ACA UCU UGA ATT and UUC AAG AUG UAG CAG UGC GTT (2) CCG UUC CCU UCU CCA UUA UTT and AUA AUG GAG AAG GGA ACG GTT. The sequences of YAP siRNA are as follows: (1) GUC AGA GAU ACU UCU UAA ATT and UUU AAG AAG UAU CUC UGA CTT (2) GUC UCA GGA AUU GAG AAC ATT and UGU UCU CAA UUC CUG AGA CTT. The sequences of GNA11 siRNA are as follows: ACU UGU AGA GGA UCU UGA GCG and CUC AAG AUC CUC UAC AAG UAC. The sequences of GNAQ were UUA UCU UCA UCA GAG UAU CCU and GAU ACU CUG AUG AAG AUA AAA. The negative control siRNA sequences were as follows: UUC UCC GAA CGU GUC ACG UTT and ACG UGA CAC GUU CGG AGA ATT. The reagent used for siRNA transduction was RNAiMAX reagent (13,778,150, Invitrogen). For lentivirus CXCR7 silencing, shCXCR7 was inserted into the insertion vector pLKO.1 and cotransfected with pMD2.G envelope plasmid and psPAX2, bound as a packaging plasmid in HEK293T cells and cotransfected to express the corresponding gene. Forty-eight hours later, the CXCR7 shRNA-expressing lentivirus solution was collected for subsequent experiments. The gene was expressed by adding 3 ml of antibiotic-free medium containing 300 µl of lentiviral suspension to MGC803 gastric cancer cells for coculture.

### Reagents

The reagents used were XMU-MP-1 (MCE, Cat. No. HY-100526), doxycycline (Dox) (Sigma, Cat. No. 33429), ACT-1004–1239 (MCE, Cat. No. HY-142617), TC14012 (MCE, Cat. No. HY-P1102), verteporfin (MCE, Cat. No. HY-B0146), GSK429286A (MCE, Cat. No. HY-11000), Y27632 (MCE, Cat. No. HY-10071), and C3 (Cytoskeleton, Inc., Cat. No. CT03).

### Immunoblot analysis

The method used to analyse relative protein expression in cells was the standard Western blotting technique. The catalogue of antibodies used for immunoblotting tests was as follows: anti-CXCR7 (ab117836, Abcam, 1:1000), anti-YAP (SC-101199, Santa Cruz, 1:1000), anti-tubulin (11,224–1-AP, Proteintech, 1:1000), anti-histone-H3 (17,168–1-AP, Proteintech, 1:1000), anti-pYAP (S127) (13,008, Cell Signaling Technology, 1:1000), anti-actin (3700, Cell Signaling Technology, 1:1000), anti-Gαq/11 (sc-392, Santa Cruz, 1:1000), anti-Gαs (sc-135914, Santa Cruz, 1:1000), anti-LATS1 (3477, Cell Signaling Technology, 1:1000), and anti-Myc (9E10, Santa Cruz, 1:1000). The protein signal was detected by an ECL kit (Millipore Co., Billerica, Massachusetts, USA).

### In vitro kinase activity assay

The phos-tag reagent Phos-tag Acrylamide (Wako, 93521) was purchased from Wako Chemicals. Gels containing phos-tag and MnCl2 (Wako, 13446–34-9) were prepared according to the manufacturer's instructions. On the prepared phos-tag gels, anti-YAP (SC-101199, Santa Cruz, 1:1000) was used for primary antibody, the YAP protein can be separated into multiple visible bands by itself according to the phosphorylation effect to observe the change in phosphorylation status.

### Quantifying cell viability

Gastric cancer cells MGC803 and Hs746T cells were treated in 12-well plates with siCXCR7 or siControl transfection or by drugs. Twenty-four hours after the cells were treated, the cells in the 12-well plates were counted, and 5000 cells were transferred to 96-well plates after counting the distillation. The number of cells was determined by measuring the relative viability of the cells at 450 nm using the CCK-8 Cell Proliferation Reagent at different time points to measure absorbance.

### Wound healing assay

In the wound healing test, MGC803 and Hs746T gastric cancer cells were transfected with siCXCR7 or siControl or treated with the indicated drugs in a 6-well plate until the cells grew without gaps, and then, a straight line was drawn along a straight edge with a 200 µl yellow sterile tip gun. Scratches of the cells were assessed by microscopic photography at the indicated time points after the line was made. The separation between the two edges was measured and compared to the distance on the first day. The recovery rate of wound healing was calculated as follows: [1—(Woundwound width at a given time/wound width at t = 0)] × 100%.

### Transwell assays

Transwell plates (8 μm pore size, Corning) were used for cell migration experiments and invasion experiments. The membranes in the upper chamber of the plates were coated with a special matrix gel (BD Biocoat, USA). After treatment with the appropriate siRNA or drugs, the cells were fixed on the membrane bottom with 4% paraformaldehyde for 10 min. After fixation, they were washed three times with water. Subsequently, the cells were stained with crystal violet for 10 min, followed by another three washes with water. Finally, cell counting was performed under a microscope using a 20 × objective. Each experiment was conducted in triplicate.

### Immunofluorescence (IF) staining

MGC803 and Hs746T cells were added to 24-well plates, treated with small molecule reagents after they were plated, and transferred to small dialysis slides after 24 h. After they were plated, gastric cancer cells were fixed on bottom coverslips using 4% paraformaldehyde and stained with anti-CXCR7 (ab117836, Abcam, 1:200) and anti-YAP (14,074, Cell Signaling Technology, 1:200) primary antibodies for one hour at room temperature. After that, the cells were washed with PBS. Cells were then incubated again with fluorophore-conjugated secondary antibody (Invitrogen, Carlsbad, CA). After PBS washes, the cells were stained for nuclei with the cell nuclear dye DAPI (Life Technology). After the cells reacted and were washed, images of staining for cell-specific proteins were obtained with a confocal laser scanning microscope (Leica TCS SP8 STED) after dropping in anti-quenching reagent. The stained images taken were integrated into a single image using ImageJ software, and their fluorescence intensity was measured for analysis.

### Immunoprecipitation

Western blotting was conducted as described in previously published studies [16, 31]. Total lysates of MGC803 and Hs746T gastric cancer cells were preincubated with rabbit IgG for 1 h. We used Cell lysis buffer for Western and IP (Beyotime, P0013).It contained 20 mM Tris(pH7.5), 150 mM NaCl, 1% Triton X-100. sodium pyrophosphate, β-glycerophosphate, EDTA, Na3VO4, leupeptin with Protease inhibitor Cocktail (TargetMol, C0001). After removal of IgG as a negative control, immunoprecipitation was performed with anti-LATS1 (3477, Cell Signaling Technology, 1:1000), and antibodies were incubated with protein for 4 h at 4 °C, with rabbit IgG (Santa Cruz) used as a negative control. Afterwards, protein A was added for the corresponding binding of beads to antibodies, and finally, western blot loading buffer was added. After lysis, the cell solution was detected with anti-CXCR7 (ab117836, Abcam, 1:1000) or anti-YAP (SC-101199, Santa Cruz, 1:1000) for the presence or absence of bound proteins.

### Colony formation assay

Gastric cancer cells MGC803 and Hs746T cells were spread in 12-well plates and treated with CXCR7 siRNA or siControl or the indicated concentrations of chemical reagents after they were allowed to grow against the wall. Twenty-four hours later, the cells were suspended in trypsin, and cell counting was performed (5,00 cells/well in six-well plates). The cells were cultured for two weeks after adding sufficient medium, and the medium was changed every three days with fresh medium containing serum. After they grew to the appropriate density, the cells were fixed with 4% paraformaldehyde for ten minutes, washed with PBS and then stained with crystal violet staining solution for the fixed colonies. Colonies were counted by counting specific areas of each condition, and the counts were compared.

### Extreme limiting dilution assay and self-renewal capacity

Control or experimental cells were inoculated into ultralow adherence 96-well plates (Corning, CLS3474) at different cell doses and incubated for 10 days at 37 °C in standard pH under sphere-forming conditions. Decreasing numbers (200–100-50–20 cells per well) of cells were cultured in medium plus 20 ng/ml basic fibroblast growth factor (bFGF) (R&D Systems, 233-FB-010) and 20 ng/ml epidermal growth factor (EGF) (R&D Systems, 236-EG-200). Colony formation was assessed by visual observation. For each dilution series, we counted the wells that showed sphere formation on Day 11. Data were analysed and displayed using the Extreme Limiting Dilution Assay (ELDA) software, which can be downloaded at: https://bioinf.wehi.edu.au/software/elda/ [32].

### Flow cytometry analysis

In cell cycle analysis experiments, MGC803 and Hs746T cells were synchronized at G0/G1 by serum starvation. MGC803 and HS746T cells were seeded in 12-well plates and transfected with siCXCR7 or siControl or the appropriate concentration of chemical reagents specified with 10% FBS and left to react for 24 h or treated with the appropriate chemical reagents for the specified time. The suspended cells were first collected with trypsin, followed by fixation treatment with 70% ethanol, and then propidium iodide was used to stain the cells. In addition, the percentage of cells in each phase of the cell cycle was analysed by Modfit software. In the apoptosis assay, the cells were treated with the appropriate reagents. After that, the cells were stained with propidium iodide and Annexin V. The proportion of each cell phase was determined.

### Luciferase reporter assay

Gastric cancer MGC803 and Hs746T cells were cotransfected and plated with siCXCR7 or siControl or with appropriate chemistry and TEAD luciferase reporter vector, and the cells were collected after 24 h by digestion with trypsin and then assayed. A dual luciferase assay kit (Promega) was used for this experiment. The pRL-null vector (Promega) expressing Renilla luciferase was an internal control.

### In vivo tumorigenesis assay

For the in vivo tumorigenesis assay in mice, MGC803 cells were inoculated with the corresponding lentiviral cells. After 48 h of infiltration, the cells were screened for resistant target cells by incubation with 0.5 μg/ml puromycin for three days. Gastric cancer cells MGC803 cells (2 × 10^6^) of the screened stable transgenic strain were injected into the backs of 4-week-old female BALB/c nude mice. Alternatively, mice were treated with the drug after cell injection, and tumour formation in nude mice was monitored for 4 weeks. Tumour volume = 0.5 × length × width^2.^ Mice were sacrificed 5 weeks after injection. The weight of their removed tumours was calculated, and photographs were taken. Mice were purchased from SPF (Beijing) Biotechnology Co., Ltd. This experiment was conducted according to the protocol approved by the ethics commission of the Second Hospital of Shandong University. Approval Protocol Number KYLL-2023LW030.

### Tissue microarray (TMA) and immunohistochemistry (IHC) analysis

Ninety paraffin-embedded gastric cancer samples were received from Shanghai Odo Biotech (http://www.superchip.com.cn). Approval Protocol Number: HStmA180Su11. The samples were inspected by pathologists. Pathological grade and lymph node metastasis conditions were acquired for each sample. The utilization of the samples was sanctioned by the company, and written informed consent was obtained from all patients. Samples were stained using antibodies specific for CXCR7 (Abcam, 72,100) and YAP (Cell Signaling Technology, 14,074) and assessed according to the Fromowitz criteria. The staining was graded in intensity as follows: no positive staining, 0; weak positive staining, 1; intermediate positive staining, 2; and high positive staining, 3. The percentage of positive cells was categorized into four categories: staining percentage of 0–25%, 1; staining percentage of 26–50%, 2; staining percentage of 51–75%, 3; and staining percentage of 76–100%, 4. Staining scores of 1–2 was regarded as indicating low expression, while staining scores of 3–4 was regarded as indicating high expression. At least three regions were counted per core region.

### ChIP assay

The ChIP (chromatin immunoprecipitation) assay was conducted following to the methodology outlined in a previously published study [33]. In brief, cells were crosslinked by adding formaldehyde to a final concentration of 1% for 10 min only or 2 mM DSG crosslinker (CovaChem, Cat. No.13301) at room temperature for 1 h followed by secondary fixation with 1% formaldehyde (Pierce, Cat. No. 28908) for 10 min and quenched by adding glycine. Subsequently, cells were washed with PBS and subject to cell lysis. Cell lysis buffer contains SDS Lysis Buffer (Beyotime P2078-11), Phosphatase Inhibitor Cocktail (TargetMol, Cat: C0002) and Protease Inhibitor Cocktail (TargetMol, Cat: 0001). The cell extracts were subject to sonication. To achieve chromatin fragmentation, sonication was employed using a Bioruptor sonicator (Diagenode). ChIP assay involved sonication of chromatin fragments, resulting in fragments typically ranging from approximately 100 to 500 base pairs (bp) in size. The sonication parameters were set as follows: Power: Typically set 25%, adjust as needed depending on the sample type. Sonication Cycle: 30 s on, 30 s off (pulse mode), repeated 10–20 times. Amplitude: High setting. Temperature: Samples were maintained in an ice-water bath during sonication to prevent overheating. After centrifugation, the cell extracts were incubated with prepared YAP antibody (CST, Cat. No. 14074), washed in wash buffer for five times and decross-linked ChIP in elution buffer at 65 °C overnight. Quantitative PCR analysis was performed with a DNA extraction kit (Qiagen, Cat. No. 28106). ChIP-qPCR analysis was performed with the following primer sequences: CTGF F: TGT GCC AGC TTT TTC AGA CG, R: TGA GCT GAA TGG AGT CCT ACA CA; CYR61 F: AGC AAA CAG CTC ACT GCC TT, R: ATG GTA GTT GGA GGG TCG TG; CXCR7: F: CAG GAA CAG AGA GAG CCA GC, R: ATT GAG AGG CCC AGG GTT G.

### RNA sequencing and data analysis

The RNA sequence data were stored in the Gene Expression Repository (GEO) (GSE233094). Genes with differential representation (*P* < 0.05 and fold change > 2) were analysed by Ingenuity Pathway Analysis (IPA). For genomic rich analysis of RNA-seq data, the conserved Hippo signature gene set was utilized and downloaded from Molecular Signatures Database v7.4. Volcano plots were created with the 'ggplot2' package in R.

### TCGA data and analysis of survival data

Gene exposure databases of 376 gastric cancer patients were obtained from the TCGA database. The correlation between CXCR7 expression and patient survival in gastric cancer was generated using GEPIA online software; GSEA online software was used to generate the results using default parameters. Volcano plots were generated with the 'ggplot2' package in R (threshold *P* < 0.05, equivalent change > 1.5).

### Statistical analysis

Data were analysed using GraphPad Prism 8 software or SPSS version 23. Data are presented as the mean ± s.e.m. values. Variations among each individual set were appraised with Student’s t test. Survival analysis was performed by the Kaplan‒Meier method and log-rank test. A difference was assumed to be statistically significant when *P < 0.05 (**P < 0.01; ***P < 0.001).

## Results

### CXCR7 correlates with the gene signature of the Hippo pathway in gastric cancer

**Fig. 1:**
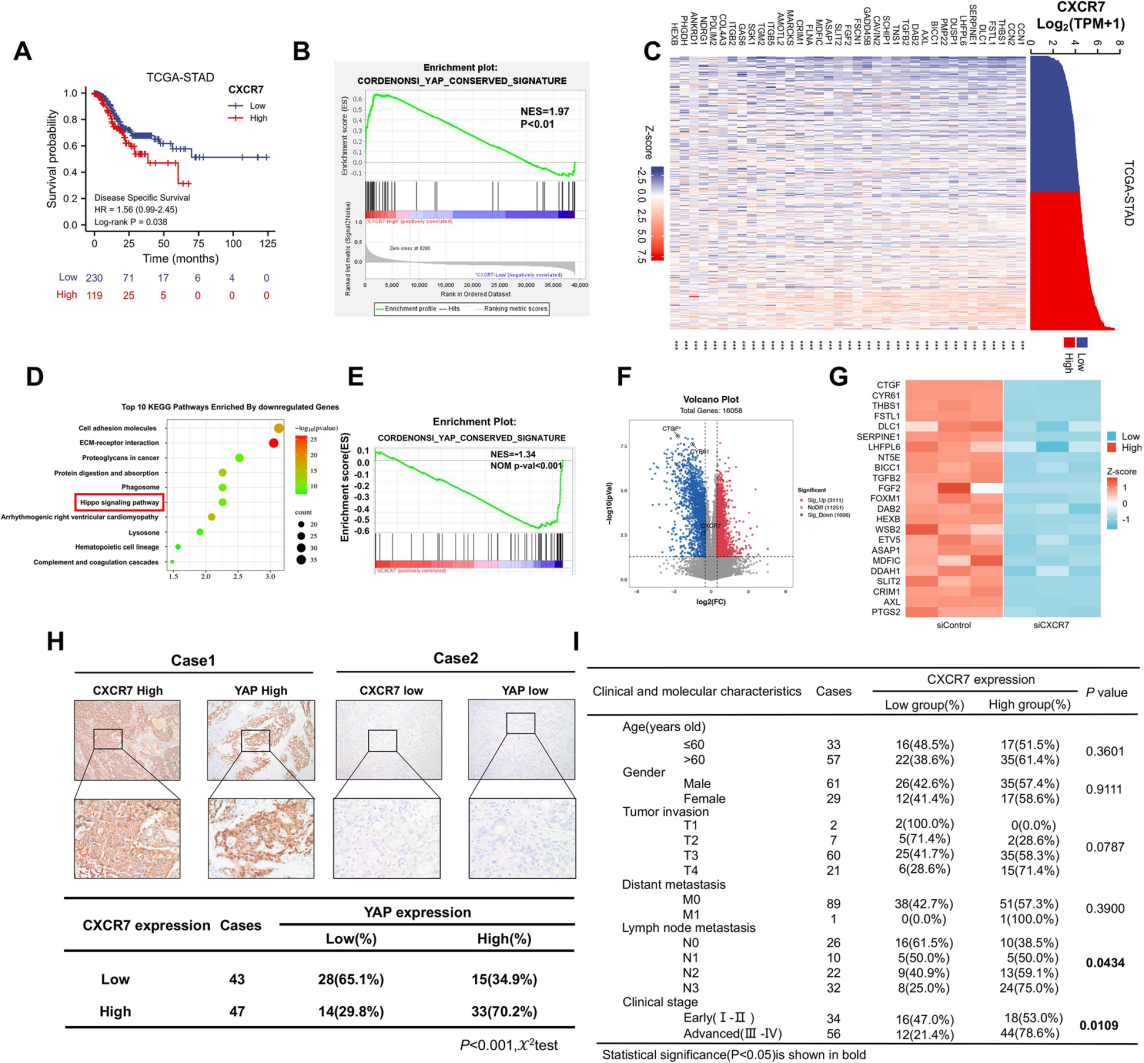
CXCR7 correlates with the gene signature of the Hippo pathway in gastric cancer. **A** TCGA data analysis showed that the CXCR7 expression level correlated with poor survival in gastric cancer patients. **B** GSEA of TCGA data showed a significant positive correlation between CXCR7 and the YAP target gene signature (https://tcga-data.nci.nih.gov). **C** TCGA database analysis showed a significant positive correlation between the expression levels of CXCR7 and Hippo pathway target genes in gastric cancer samples.** D** RNA-seq data analysis indicated the top 10 KEGG pathways enriched by CXCR7 depletion in MGC803 cells. Threshold *P* < 0.05. **E** GSEA showed that CXCR7 depletion in MGC803 cells decreased the expression of the YAP target gene signature. **F** Volcano plot showing that CXCR7 depletion inhibits the expression of Hippo pathway signature genes (red) in MCG803 cells. Threshold criteria: *P* < 0.05 and fold change > 1.5. **G** Heatmap of YAP target genes in RNA-seq data from MGC803 cell lines treated with siControl or siCXCR7. **H** Immunohistochemical analysis of gastric cancer samples showed a positive correlation between CXCR7 and YAP (*P* < 0.001). **I** The correlation analysis revealed that CXCR7 expression correlated with lymph node metastasis and advanced tumour stage (*P* = 0.0434 and *P* = 0.0109, respectively)

We evaluated the survival effect of CXCR7 in gastric cancer samples from the TCGA database, which showed that CXCR7 expression correlated with poor survival (Fig. 1A). GSEA showed a positive correlation between CXCR7 and the YAP conserved gene signature (Fig. 1B). Since the cluster of the Hippo signature contains 37 classical target genes, the heatmap analysis showed a strong correlation between CXCR7 and each target gene (Fig. 1C). To confirm this observation, we depleted CXCR7 in MG803 cells for whole genomic expression analysis, ELISA experiment showing the concentration of CXCL12 in serum was approximately 3000 ng/mL, while the concentration of CXCL12 in the DMEM was too low to be detected (Supplementary Fig. 1A). The RNA sequencing data showed that CXCR7 depletion caused a dramatic change in the Hippo pathway (Fig. 1D), while CXCR7 depletion inhibited global Hippo target gene expression according to GSEA (Fig. 1E). The database analysis and western blot analyses show CXCR4 is not expressed in gastric cancer cells, including MGC803 and Hs746T (Supplementary Fig. 1B and C). Volcano and heatmaps indicated that a classical set of Hippo target genes was reduced, including CTGF and CYR61 (Fig. 1F, G). We further investigated the expression of CXCR7 and YAP in gastric cancer samples, and the immunohistochemistry data showed a positive correlation between YAP and CXCR7 (*P* < 0.001) (Fig. 1H, I).

### CXCR7 is required for gastric cancer cell progression

**Fig. 2:**
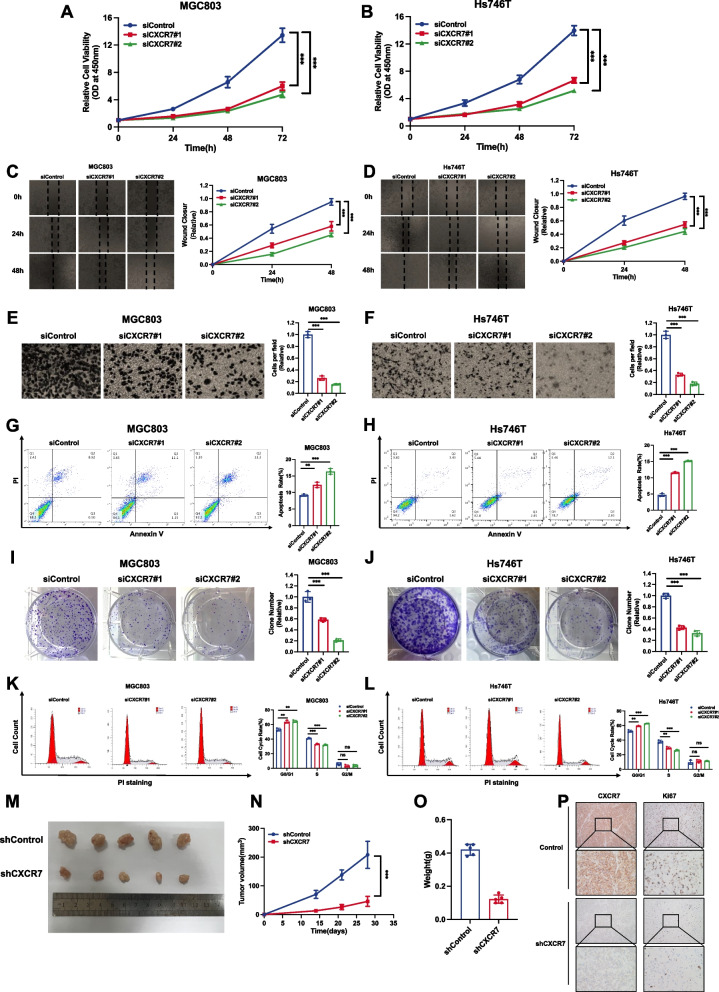
CXCR7 is required for gastric cancer cell progression. **A**, **B** Depletion of CXCR7 inhibited the proliferation of gastric cancer cells. MGC803 and Hs746T cells were transfected with siControl or siCXCR7. Two different siRNAs were used. After 24 h, CCK-8 assays were used to determine the metabolic activity of the cells at the indicated time points after transfection. Experiments were performed in triplicate. Comparison of cell growth, *P < 0.05, **P < 0.01, ***P < 0.001.**C**,** D** Wound healing assay of MGC803 and Hs746T cells transfected with siCXCR7 or siControl. Wound closure was quantified for the indicated time points. Data are expressed as the mean ± SD. **P < 0.01, ***P < 0.001 (Student's t test). **E**,** F **Depletion of CXCR7 inhibited the migration of MGC803 and Hs746T gastric cancer cells. MGC803 and Hs746T cells were transfected with siControl or siCXCR7. After 24 h, the migration was assessed by Transwell assays. Cell numbers were determined, and data are expressed as the mean ± SD. **P < 0.01, ***P < 0.001 (Student's t test). **G**, **H** Depletion of CXCR7 promoted apoptosis of MGC803 and Hs746T cells. MGC803 and Hs746T cells were transfected with siCXCR7 and siControl. After 24 h, the cells were stained with PI and Annexin V, and then, FACS analysis was performed on the cells to determine the proportion of apoptotic cells. Each group was analysed in triplicate. *P < 0.05; **P < 0.01; ***P < 0.001 for comparison. **I**, **J** Depletion of CXCR7 inhibited the colony-forming ability of MGC803 and Hs746T gastric cancer cells. MGC803 and Hs746T cells were transfected with siCXCR7 and siControl. Quantification of colony formation is shown at the indicated time points. Data are expressed as the mean ± SD. **P < 0.01, ***P < 0.001 (Student's t test). **K**, **L** Cell cycle analysis was performed to assess the effect of CXCR7 silencing on MGC803 cells and Hs746T cells. MGC803 and Hs746T cells were transfected with siCXCR7 or siControl. After 24 h, the cells were harvested, fixed in 70% ethanol and stained with propidium iodide. Cells were subjected to FACS analysis. Experiments were performed in triplicate. Comparison of cell proportions, *P < 0.05, **P < 0.01, ***P < 0.001. Representative histograms and cell cycle phase distribution plots are shown in Fig. 2 K and L, respectively. **M**–**O** Depletion of CXCR7 inhibited gastric tumour growth in vivo. MGC803 cells were stably transduced by a lentiviral vector expressing either control shRNA or CXCR7 shRNA. These MGC803 cells (2 × 10^6^) were injected into the right dorsal side of 4-week-old female BALB/c nude mice. Tumour formation in nude mice was monitored over a period of 4 weeks. Tumour volume was calculated using the following formula: tumour volume = 0.5 × length × width^2^. Five weeks after tumour cell injection, the mice were sacrificed. Tumour growth curves, weights and photographs are shown in Panels M, N and O, respectively. **P** Immunohistochemical analysis showed that CXCR7 depletion decreased the expression of Ki67 in xenograft tumours

We investigated the phenotype of CXCR7 in two gastric cancer cell lines, MGC803 and Hs746T. CXCR7 is knocked out in gastric cancer cells (Supplementary Fig. 2A). The CCK-8 assay showed that CXCR7 deletion apparently repressed gastric cancer growth (Fig. 2A and B). The wound-healing assay showed that CXCR7 silencing could hamper gastric cancer cell migration (Fig. 2C and D). In addition, the Transwell experiments demonstrated that CXCR7 depletion could significantly decrease the invasion of gastric cancer cells (Fig. 2E and F). The cell apoptosis assay showed that CXCR7 silencing increased the number of apoptotic cells in both MGC803 and Hs746T cells (Fig. 2G, H). Clonogenesis experiments also indicated that CXCR7 deletion decreased the number of clusters of MGC803 and Hs746T cells (Fig. 2I, J). ELDA experiments showed the same results (Supplementary Fig. 2B and C). Flow cytometry profiling demonstrated that CXCR7 deletion could lead to G1 phase cell cycle arrest (Fig. 2K, L). We further carried out a vivo experiment to evaluate the function of CXCR7, while the xenograft mouse model assay showed that CXCR7 silencing inhibited the potential of tumorigenesis in gastric cancer cells (Fig. 2M-O). The immunohistochemical analysis of the xenograft tumours showed decreased expression of Ki67 (Fig. 2P).

### Pharmaceutical targeting of CXCR7 restrains gastric cancer progression

**Fig. 3:**
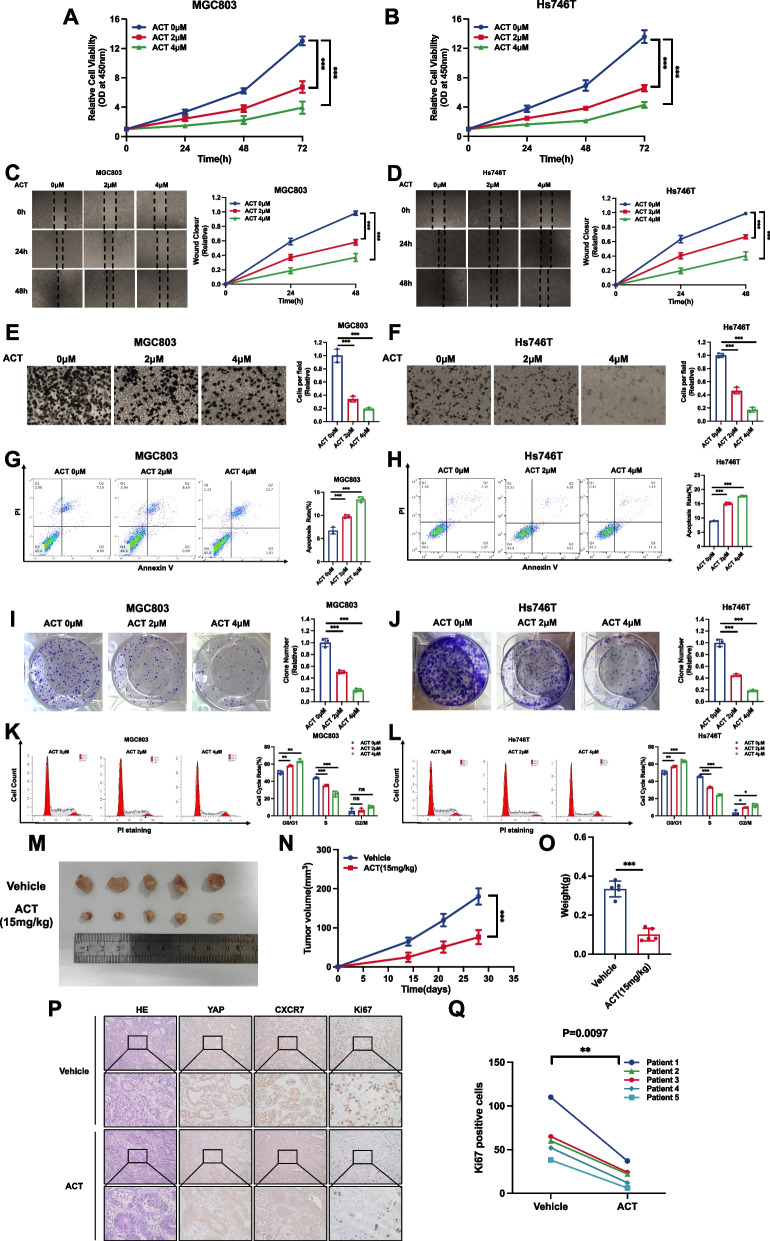
Drugs targeting CXCR7 restrain gastric cancer progression. A, B ACT antagonist treatment against CXCR7 inhibited the proliferation of gastric cancer cells. MGC803 and Hs746T cells were treated with different doses of ACT. After 24 h, CCK-8 assays were used to determine the metabolic activity of the cells at the indicated time points after transfection. Experiments were performed in triplicate. Comparison of cell growth, *P < 0.05, **P < 0.01, ***P < 0.001. **C**, **D** Wound healing assay of MGC803 and Hs746T cells treated with an antagonist against CXCR7. Wound closure was quantified for the indicated time points. Data are expressed as the mean ± SD. **P < 0.01, ***P < 0.001 (Student's t test). **E**, **F** ACT antagonist treatment against CXCR7 inhibited the migration of MGC803 and Hs746T gastric cancer cells. MGC803 and Hs746T cells were transfected with different doses of ACT. After 24 h, the migration was assessed by Transwell assays. Cell numbers were determined, and data are expressed as the mean ± SD. **P < 0.01, ***P < 0.001 (Student's t test). **G**, **H** ACT antagonist treatment against CXCR7 promoted apoptosis of MGC803 and Hs746T cells. MGC803 and Hs746T cells were transfected with different doses of ACT. After 24 h, the cells were stained with PI and Annexin V, and then, FACS analysis was performed on the cells to determine the proportion of apoptotic cells. Each group was analysed in triplicate. *P < 0.05; **P < 0.01; ***P < 0.001 for comparison. **I**, **J** ACT antagonist treatment against CXCR7 inhibited the colony-forming ability of MGC803 and Hs746T gastric cancer cells. MGC803 and Hs746T cells were transfected with different doses of ACT. Quantification of colony formation is shown at the indicated time points. Data are expressed as the mean ± SD. **P < 0.01, ***P < 0.001 (Student's t test). **K**, **L** Cell cycle analysis was performed to assess the effect of treatment with the CXCR7 antagonist ACT on MGC803 cells and Hs746T cells. MGC803 and Hs746T cells were transfected with different doses of ACT. After 24 h, the cells were harvested, fixed in 70% ethanol, and stained with propidium iodide. Cells were subjected to FACS analysis. Experiments were performed in triplicate. Comparison of cell proportions, *P < 0.05, **P < 0.01, ***P < 0.001. Representative histograms and cell cycle phase distribution plots are shown in Fig. 3K and L, respectively. **M**–**O** ACT antagonist treatment against CXCR7 inhibited gastric tumour growth in vivo. MGC803 cells (2 × 10^6^) were injected into the right dorsal side of 4-week-old female BALB/c nude mice. Tumour formation in nude mice treated with vehicle or ACT at the indicated concentrations was monitored over a period of 4 weeks. Tumour volume was calculated using the following formula: tumour volume = 0.5 × length × width^2.^ Five weeks after tumour cell injection, mice were sacrificed. Tumour growth curves, weights and photographs are shown in Panels M, N and O, respectively. **P**, **Q** In the patient-derived explant (PDEx) assay, ACT treatment inhibited the proliferation potential of gastric tumours. The gastric tumour samples were cultured ex vivo on sponges for 48 h with 10% FBS medium. The gastric tumour explants were treated with vehicle or 4 µM ACT. The samples were fixed and stained with YAP, CXCR7 and Ki67 via IHC. The Ki67-positive cells were counted for analysis

Previous studies identified ACT-1004–1239 (ACT) as an effective CXCR7 antagonist that could be utilized in inflammatory demyelinating diseases [34, 35]. We further examined the effect of ACT on the gastric cancer phenotype and Hippo signalling. Western blot experiments showed a significant increase in YAP phosphorylation levels only at the 2 µM concentration (Supplementary Fig. 3A). The CCK-8 assay showed that ACT treatment could significantly inhibit gastric cancer cell proliferation (Fig. 3A, B). The wound-healing assay showed that ACT treatment could hamper gastric cancer cell migration (Fig. 3C and D). In addition, the Transwell experiments demonstrated that ACT treatment could significantly decrease the invasion of gastric cancer cells (Fig. 3E and F). The cell apoptosis assay showed that ACT increased the number of apoptotic cells in both MGC803 and Hs746T cells (Fig. 3G, H). The colony formation assay also showed that ACT treatment could decrease the number of clones for both MGC803 and Hs746T cells (Fig. 3I, J). ELDA experiments showed the same results (Supplementary Fig. 3B and C). Flow cytometry analysis showed that ACT treatment caused G1-phase cell cycle arrest (Fig. 3K, L). We further carried out an in vivo experiment to evaluate the effect of ACT, while the xenograft mouse model assay showed that ACT inhibited the potential of tumorigenesis in gastric cancer cells (Fig. 3M-O). We further utilized the ex vivo culture model of primary gastric cancer samples while allowing the evaluation of drug effects and maintaining the native tissue architecture. The obtained gastric tumours were cut into several parts and treated with 4 µM ACT for in vitro growth on sponges. In the patient-derived explant assay, immunohistochemical analysis revealed a pronounced reduction in nuclear YAP localization, indicating the inhibition of YAP nuclear translocation induced by ACT, while we found that ACT dramatically inhibited the level of the proliferation marker Ki67 (Fig. 3P, Q).

### Pharmaceutical activation of CXCR7 promotes gastric cancer progression

**Fig. 4:**
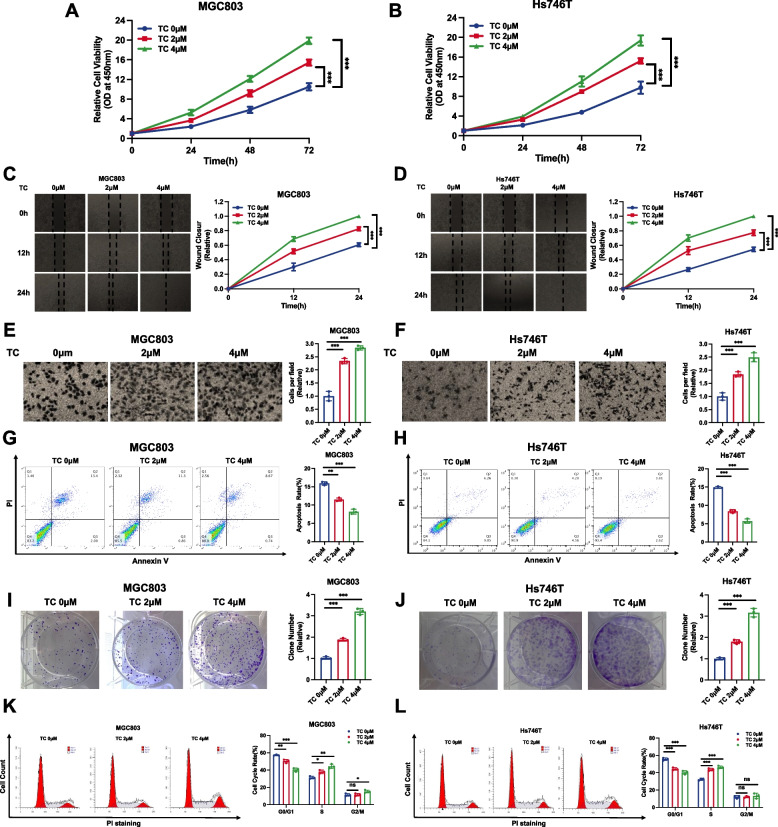
Drugs activating CXCR7 promote gastric cancer progression. **A**, **B** CXCR7 activation via TC promoted the proliferation of gastric cancer cells. MGC803 and Hs746T cells were treated with different doses of TC. After 24 h, CCK-8 assays were used to determine the metabolic activity of the cells at the indicated time points after transfection. Experiments were performed in triplicate. Comparison of cell growth, *P < 0.05, **P < 0.01, ***P < 0.001. **C**, **D** Wound healing assay of MGC803 and Hs746T cells with CXCR7 activation via TC. Wound closure was quantified for the indicated time points. Data are expressed as the mean ± SD. **P < 0.01, ***P < 0.001 (Student's t test). **E**, **F** CXCR7 activation via TC promotes the migration of MGC803 and Hs746T gastric cancer cells. MGC803 and Hs746T cells were transfected with different doses of TC. After 24 h, the migration was assessed by Transwell assays. Cell numbers were determined, and data are expressed as the mean ± SD. **P < 0.01, ***P < 0.001 (Student's t test). **G**, **H** CXCR7 activation via TC inhibited apoptosis of MGC803 and Hs746T cells. MGC803 and Hs746T cells were transfected with different doses of TC. After 24 h, the cells were stained with PI and Annexin V, and then, FACS analysis was performed on the cells to determine the proportion of apoptotic cells. Each group was analysed in triplicate. *P < 0.05; **P < 0.01; ***P < 0.001 for comparison. **I**,** J** CXCR7 activation via TC promotes the colony-forming ability of MGC803 and Hs746T gastric cancer cells. MGC803 and Hs746T cells were transfected with different doses of TC. Quantification of colony formation is shown at the indicated time points. Data are expressed as the mean ± SD. **P < 0.01, ***P < 0.001 (Student's t test). **K**, **L** Cell cycle analysis was performed to assess the effect of CXCR7 activation via TC on MGC803 cells and Hs746T cells. MGC803 and Hs746T cells were transfected with different doses of TC. After 24 h, the cells were harvested, fixed in 70% ethanol and stained with propidium iodide. Cells were subjected to FACS analysis. Experiments were performed in triplicate. Comparison of cell proportions, *P < 0.05, **P < 0.01, ***P < 0.001. Representative histograms and cell cycle phase distribution plots are shown in Fig. 4 K and L, respectively

We further utilized the specific CXCR7 agonist TC14012 (TC) in gastric cancer phenotypes [36, 37]. Western blot experiments showed a significant decrease in YAP phosphorylation levels only at the 2 µM concentration (Supplementary Fig. 4A). The CCK-8 assay showed that TC treatment could significantly promote gastric cancer proliferation (Fig. 4A, B). The wound-healing assay showed that TC treatment could facilitate gastric cancer cell migration (Fig. 4C and D). In addition, the Transwell experiments demonstrated that TC treatment could significantly enhance the invasion of gastric cancer cells (Fig. 4E and F). The cell apoptosis assay showed that ACT decreased the number of apoptotic cells in both MGC803 and Hs746T cells (Fig. 4G, H). The clone formation assay also showed that TC treatment could increase the number of clones for both MGC803 and Hs746T cells (Fig. 4I, J). ELDA experiments showed the same results (Supplementary Fig. 4B and C). Flow cytometric experimental analysis revealed that ACT treatment reduced the ratio of cells in G1 stages (Fig. 4K, L). All these data prove the supportive effects of CXCR7 on gastric cancer growth.

### CXCR7 activates the YAP axis by inducing YAP dephosphorylation

**Fig. 5:**
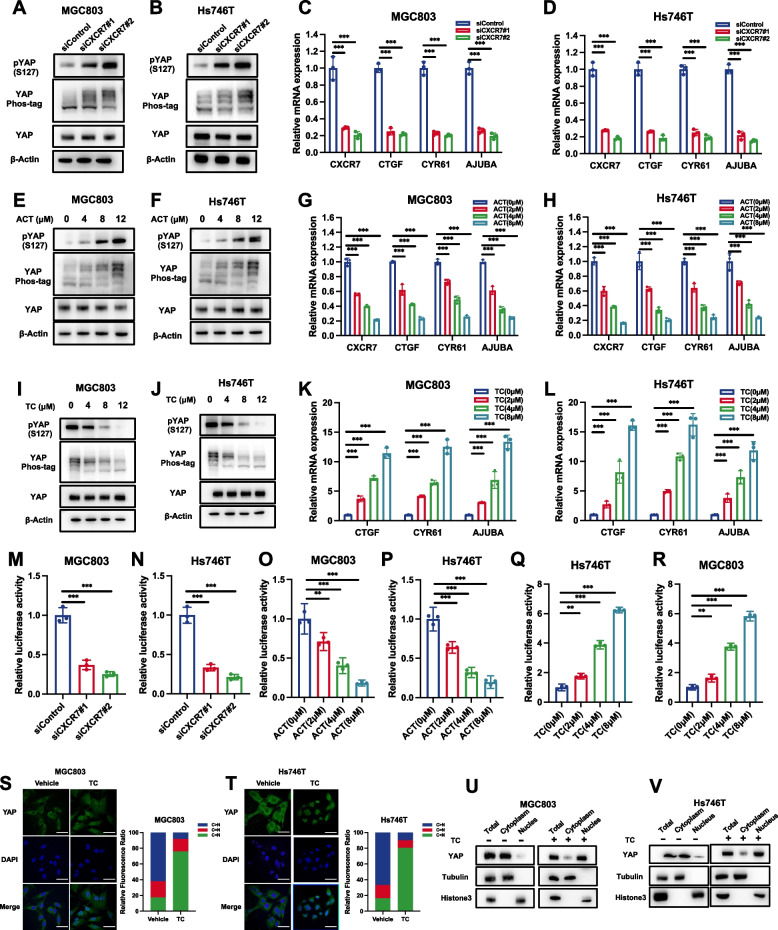
CXCR7 activates the YAP axis by inducing YAP dephosphorylation. **A**, **B** CXCR7 depletion in MGC803 and Hs746T cells induced YAP phosphorylation as determined by immunoblotting with S127 phospho-antibody and phospho-tag assay. MGC803 and Hs746T cells were transfected with different siCXCR7s. Immunoblotting was performed with the indicated antibodies. Phos-tagged gels were used to assess the phosphorylation status of YAP. **C**, **D** CXCR7 depletion decreased Hippo target gene expression in MGC803 and Hs746T cells. MGC803 and Hs746T cells were transfected with siControl or siCXCR7. After 48 h, total RNA was extracted for gene expression analysis. Each group was tested in triplicate. *P < 0.05, **P < 0.01, ***P < 0.001 for comparisons of target gene expression. **E, F** Antagonist ACT treatment against CXCR7 induced YAP phosphorylation as determined by immunoblotting with S127 phospho-antibody and phospho-tag assay. MGC803 and Hs746T cells were transfected with different doses of ACT as indicated. Immunoblotting was performed with the indicated antibodies. Phos-tagged gels were used to assess the phosphorylation status of YAP. **G**, **H** ACT antagonist treatment against CXCR7 decreased Hippo target gene expression in MGC803 and Hs746T cells. MGC803 and Hs746T cells were treated with different doses of ACT as indicated. Total RNA was extracted for gene expression analysis. Each group was tested in triplicate. *P < 0.05, **P < 0.01, ***P < 0.001 for comparisons of target gene expression. **I**, **J** CXCR7 activation via TC induced YAP dephosphorylation as determined by immunoblotting with S127 phospho-antibody and phospho-tag assay. MGC803 and Hs746T cells were transfected with different doses of TC as indicated. Immunoblotting was performed with the indicated antibodies. Phos-tagged gels were used to assess the phosphorylation status of YAP. **K**, **L** CXCR7 activation via TC increased Hippo target gene expression in MGC803 and Hs746T cells. MGC803 and Hs746T cells were treated with different doses of TC as indicated. Total RNA was extracted for gene expression analysis. Each group was tested in triplicate. *P < 0.05, **P < 0.01, ***P < 0.001 for comparisons of target gene expression. **M**, **N** CXCR7 depletion in MGC803 and Hs746T cells decreased TEAD response element activity. MGC803 and Hs746T cells were transfected with siControl or siCXCR7. After 24 h, the cells were transfected with TEAD luciferase reporter plasmids. After another 24 h, the cells were harvested for luciferase activity analysis. **O**, **P** CXCR7 blockage via ACT in MGC803 and Hs746T cells decreased TEAD response element activity. MGC803 and Hs746T cells were transfected with different doses of ACT as indicated. After 24 h, the cells were transfected with TEAD luciferase reporter plasmids. After another 24 h, the cells were harvested for luciferase activity analysis. **Q**, **R** CXCR7 activation via TC increased TEAD response element activity. MGC803 and Hs746T cells were transfected with different doses of TC as indicated. After 24 h, the cells were transfected with TEAD luciferase reporter plasmids. After another 24 h, the cells were harvested for luciferase activity analysis. **S**, **T** TC promotes YAP translocation from the cytoplasm to the nucleus. MGC803 and Hs746T cells were stimulated with different doses of TC as indicated. Endogenous YAP (green) and nuclei (blue) were stained with specific antibodies and DAPI, respectively; scale bar, 20 mm. Quantifications of YAP subcellular localization from at least 100. C, cytoplasm; N, nucleus. **U**, **V** Nucleoplasm separation experiments by immunoblotting confirmed that TC induced YAP protein translocation from the cytoplasm to the nucleus in MGC803 and Hs746T cells

Since CXCR7 belongs to the GPCR protein family [38], we further investigated the role of YAP in modulating CXCR7 signalling and tested whether CXCR7 could activate YAP in gastric cancer cells. CXCR7 is knocked out in gastric cancer cells (Supplementary Fig. 5A). CXCR7 depletion in MGC803 and Hs746T cells induced YAP phosphorylation, as determined by immunoblotting with an S127 phospho-antibody and phospho-tag assay (Fig. 5A and B). We tested the mRNA levels of YAP target genes in MGC803 and Hs746T cells, which showed that the depletion of CXCR7 could inhibit the expression of target genes, including CTGF and CYR61 (Fig. 5C and D). Blockage of CXCR7 by ACT showed a similar effect in gastric cancer cells. ACT treatment increased the phosphorylation level of YAP and decreased YAP target gene expression in MGC803 and Hs746T cells (Fig. 5E-H). Consistently, the activation of CXCR7 via TC showed enhanced activity of the YAP axis. Treatment with TC decreased the phosphorylation level of YAP and increased YAP target gene expression in MGC803 and Hs746T cells (Fig. 5I-L). Western blot simultaneously shows the effects of ACT and TC on YAP phosphorylation (Supplementary Fig. 5B). The luciferase reporter experiment showed that CXCR7 depletion or blockage in MGC803 and Hs746T cells decreased TEAD response element activity, while CXCR7 activation via TC enhanced TEAD response element activity (Fig. 5M-R). Since the dephosphorylation of YAP could cause its nuclear export and cytoplasmic retention, we further examined the localization of YAP. The immunostaining data showed that CXCR7 activation via TC could induce the nuclear localization of YAP (Fig. 5S, T), which was further strengthened via a nuclear-cytosol separation assay (Fig. 5U, V). The further experiments implicated that silencing CXCR7 or adding the CXCR7 inhibitor ACT led to a modest reduction in TAZ protein levels, while the addition of a CXCR7 agonist could slightly increase TAZ protein (Supplementary Fig. 5C-E). To further confirm the phosphorylation status of TAZ, we performed phos-tag experiments, which revealed an increase in TAZ phosphorylation levels upon CXCR7 inhibition, while a decrease of TAZ phosphorylation upon CXCR7 activation (Supplementary Fig. 5C-E). These findings aligned with the phosphorylation levels observed for YAP and indicated that CXCR7 could also induce the de-phosphorylation of TAZ.

### CXCR7 facilitates gastric cell progression via the YAP axis

**Fig. 6:**
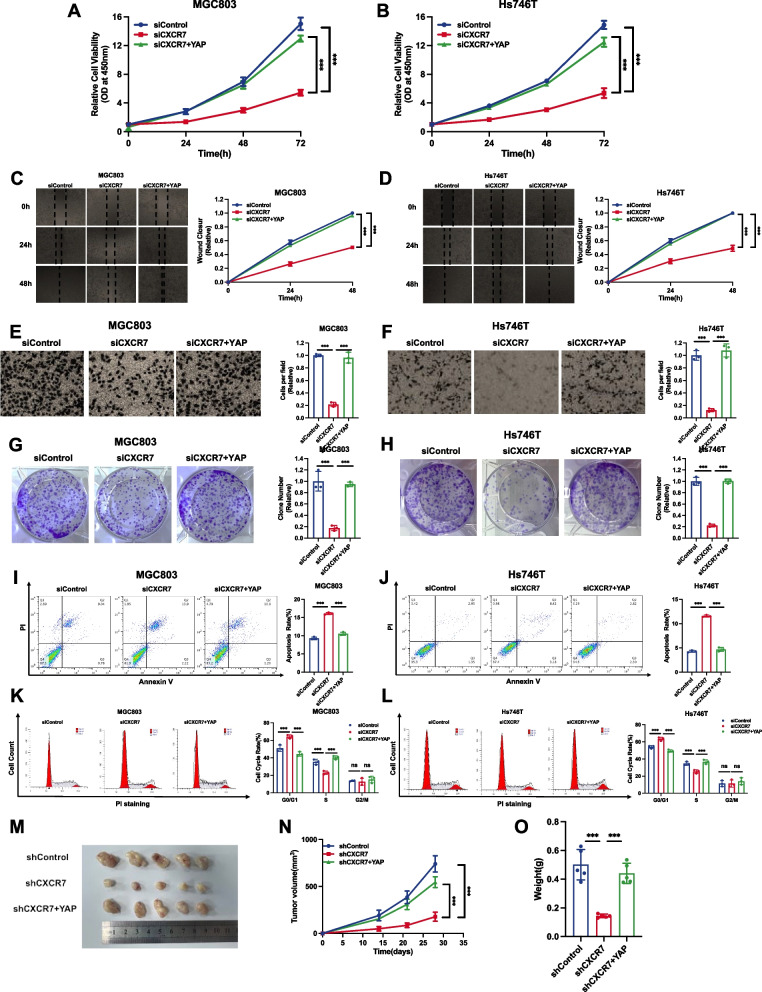
CXCR7 facilitates gastric cancer progression via the Hippo/YAP axis. **A**, **B** Depletion of CXCR7 inhibited the proliferation of gastric cancer cells, which was partially rescued by YAP overexpression. MGC803 and Hs746T cells were transfected with siControl or siCXCR7. After 24 h, the cells were transfected with YAP plasmid or empty vector. After 48 h, CCK-8 assays were used to determine the metabolic activity of the cells at the indicated time points after transfection. Experiments were performed in triplicate. Comparison of cell growth, *P < 0.05, **P < 0.01, ***P < 0.001. **C, D** Wound healing assays of MGC803 and Hs746T cells transfected with siCXCR7 or siControl, and this effect was reversed by YAP overexpression. Wound closure was quantified for the indicated time points. Data are expressed as the mean ± SD. **P < 0.01, ***P < 0.001 (Student's t test). **E**, **F** Depletion of CXCR7 inhibited the migration of MGC803 and Hs746T gastric cancer cells, and this effect was reversed by YAP overexpression. MGC803 and Hs746T cells were transfected with siControl or siCXCR7. After 24 h, cells were transfected with YAP plasmid or empty vector. After 48 h, the migration was assessed by Transwell assays. Cell numbers were determined, and data are expressed as the mean ± SD. **P < 0.01, ***P < 0.001 (Student's t test). **G**, **H** Depletion of CXCR7 promoted apoptosis of MGC803 and Hs746T cells, and this effect was reversed by YAP overexpression. MGC803 and Hs746T cells were transfected with siCXCR7 and siControl. After 24 h, the cells were transfected with YAP plasmid or empty vector. After 48 h, cells were stained with PI and Annexin V, and then, FACS analysis was performed on the cells to determine the proportion of apoptotic cells. Each group was analysed in triplicate. *P < 0.05; **P < 0.01; ***P < 0.001 for comparison. **I**, **J** Depletion of CXCR7 inhibited the colony-forming ability of MGC803 and Hs746T gastric cancer cells, and this effect was reversed by YAP overexpression. MGC803 and Hs746T cells were transfected with siCXCR7 and siControl. After 24 h, the cells were transfected with YAP plasmid or empty vector. Quantification of colony formation is shown at the indicated time points. Data are expressed as the mean ± SD. **P < 0.01, ***P < 0.001 (Student's t test). **K**, **L** Cell cycle analysis was performed to assess the effect of CXCR7 silencing on MGC803 and Hs746T cells, and this effect was reversed by YAP overexpression. MGC803 and Hs746T cells were transfected with siCXCR7 or siControl. After 24 h, the cells were transfected with YAP plasmid or empty vector. After 48 h, cells were harvested, fixed in 70% ethanol, and stained with propidium iodide. Cells were subjected to FACS analysis. Experiments were performed in triplicate. Comparison of cell proportions, *P < 0.05, **P < 0.01, ***P < 0.001. Representative histograms and cell cycle phase distribution plots are shown in Fig. 6 K and L, respectively. **M**–**O** Representative image of tumours derived from BALB/c nude mice injected with the indicated stably transfected MGC803 cells. These MGC803 cells (2 × 10^6^) were injected into the right dorsal side of 4-week-old female BALB/c nude mice. Tumour formation in nude mice was monitored over a period of 4 weeks. Tumour volume was calculated using the following formula: tumour volume = 0.5 × length × width^2^. Five weeks after tumour cell injection, mice were sacrificed. Tumour growth curves, weights and photographs are shown in Panels M, N and O, respectively

To explore the logical link between the Hippo pathway and gastric cancer in CXCR7 function, we carried out a further rescue assay. The CCK-8 assay indicated that CXCR7 depletion inhibited the proliferation of gastric cancer cells, which could be recovered by further YAP overexpression (Fig. 6A, B). Wound healing experiments indicated that CXCR7 deprivation significantly restrained the migration of gastric cancer cells, and this effect could be in part rescued by further YAP overexpression (Fig. 6C, D). In the Transwell experiment, the deletion of CXCR7 inhibited the invasive ability of gastric cancer cells, and these effects could be partially rescued by further YAP overexpression (Fig. 6E, F). In the clonogenesis test, CXCR7 deletion reduced the clonogenic number of gastric cancer cells, which was further augmented by YAP overexpression (Fig. 6G, H). ELDA experiments showed the same results (Supplementary Fig. 6A and B). In the FACS analysis, CXCR7 depletion increased cell apoptosis in gastric cancer cells, while further YAP overexpression partially rescued the number of apoptotic cells (Fig. 6I, J). Cell cycle analysis showed that CXCR7 depletion could significantly increase the proportion of cells in G1 phase, which could be partially rescued by further YAP overexpression (Fig. 6K, L). Cell cycle assays showed that CXCR7 knockdown markedly increased the fraction of cells in the G1 stage, and this increase was partially rescued by the further overexpression of YAP (Fig. 6K, L). A xenograft mouse model showed that CXCR7 depletion could arrest gastric cancer tissue tumour development in MGC803 cells, while further YAP overexpression could partially rescue the growth arrest induced by CXCR7 silencing (Fig. 6M-O).

### CXCR7 activates YAP through the Gα_q/11_-ROCK-LATS axis in gastric cancer

**Fig. 7:**
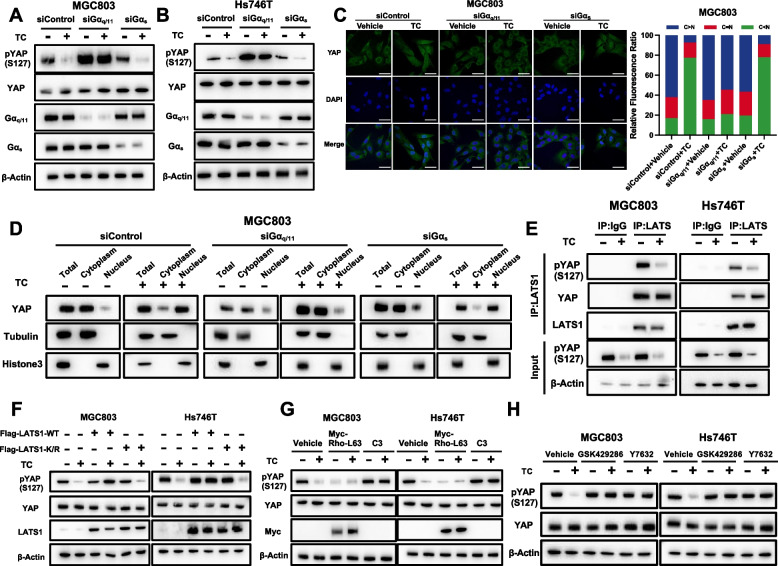
CXCR7 activates YAP through the Gα_q/11_-ROCK-LATS axis in gastric cancer. **A**, **B** CXCR7 activation induced YAP dephosphorylation through Gaq/11. MGC803 and Hs746T cells were transiently transfected with control, Ga_q/11_, or Ga_s_ siRNAs. MGC803 and Hs746T cells were treated with TC. YAP, phosphorylated YAP, Ga_q/11_ and Ga_s_ were determined by immunoblotting. **C** CXCR7 activation induces YAP nuclear localization through Ga_q/11_. MGC803 cells were transiently transfected with control, Ga_q/11_ or Ga_s_ siRNAs. MGC803 cells were treated with TC. Endogenous YAP (green) and nuclei (blue) were stained with specific antibodies and DAPI, respectively; scale bar, 20 mm. Quantifications of YAP subcellular localization from at least 100 randomly selected cells. C, cytoplasm; N, nucleus. **D** Nucleoplasm separation experiments by immunoblotting confirmed that Gα_q/11_ silencing could block the YAP nuclear accumulation caused by CXCR7 activation in MGC 803 cells. **E** CXCR7 activation via TC decreases LATS1 activity. MGC803 and Hs746T cells were stimulated with TC. LATS1 was immunoprecipitated. Phosphorylation of YAP by LATS1 was determined by a phospho-YAP antibody. **F** Ectopic expression of LATS1 blocks YAP dephosphorylation induced by CXCR activation. MGC803 and Hs746T cells were transiently transfected with control, LATS1 wild type (WT), or kinase dead mutant (K/R). MGC803 and Hs746T cells were treated with TC for 1 h. Phosphorylation and protein levels of YAP were determined by immunoblotting. **G** Rho GTPase is involved in YAP dephosphorylation induced by CXCR activation. MGC803 and Hs746T cells were transiently transfected with control, Myc-Rho-L63, or C3. MGC803 and Hs746T cells were treated with TC. Total YAP and phosphorylated YAP protein levels were determined by immunoblotting. **H** ROCK is required for CXCR7-induced YAP activation. Serum-starved MGC803 and Hs746T cells were pretreated with GSK429286 (1 mmol/L) or Y27632 (1 mmol/L) for 4 h, followed by treatment with TC. Total YAP and phosphorylated YAP protein levels were determined by immunoblotting

Since CXCR7 is a member of the GPCR family, activation might occur through several trimeric G proteins, such as Gα_q/11_ and Gα_s_ [38, 39]. To determine which Gα protein was involved in YAP regulation by CXCR7, we silenced Gα_q/11_ or Gα_s_ in gastric cancer cells. Depletion of Gα_q/11_ significantly blocked the dephosphorylation of YAP induced by TC, while depletion of Gα_s_ had little effect in MG803 and Hs746T cells (Fig. 7A, B). Besides, we did another rescue assay in which showed that CXCR7 depletion could increase yap phosphorylation, while Gaq11 activation could diminish such yap phosphorylation change, but Gs does not have such effect (Supplementary Fig. 7A). We are safe to draw the conclusion that CXCR7 exerts its function on YAP phosphorylation via Gaq11, but not Gs. Consistently, TC-induced YAP nuclear localization could be partially blocked by Gα_q/11_ silencing in MGC803 cells, but Gαs depletion had little effect (Fig. 7C). We further carried out a nuclear-cytoplasm separation assay, which showed that Gα_q/11_ silencing could block the YAP nuclear accumulation caused by TC induction in MGC 803 cells (Fig. 7D). Since LATS1/2 are the major kinases responsible for GPCR proteins in Hippo signalling [40, 41], we investigated whether LATS is involved in YAP regulation by CXCR7. We measured the kinase activity of LATS1 immunoprecipitated from MGC803 and Hs746T cells. CXCR7 activation by TC treatment inhibited LATS1 kinase activity, which was coincident with YAP dephosphorylation (Fig. 7E). In addition, overexpression of the WT form of LATS1, but not the kinase-dead form of LATS1, blocked the dephosphorylation effect of YAP caused by CXCR7 activation (Fig. 7F). The Rho/ROCK pathway is an important downstream signalling pathway in response to GPCR activation and an important upstream pathway of the Hippo pathway [42, 43]. We further examined whether Rho GTPase is involved in YAP activation by CXCR7. Overexpression of Rho-L63, which is the active mutant of Rho, could significantly dephosphorylate YAP, regardless of CXCR7 activation. However, botulinum toxin C3, which is an inhibitor of Rho GTPase [44], blocked the dephosphorylation effect of YAP caused by CXCR7 activation (Fig. 7G). We further examined whether the Rho-associated protein kinases (ROCK) are involved in modulating the Hippo pathway by CXCR7, and we tested two inhibitors of ROCK (GSK429286 and Y27632) [45, 46]. Immunoblotting showed that ROCK inhibition strongly suppressed YAP dephosphorylation, which was induced by CXCR7 activation. The further findings indicated that silencing CXCR7 did not alter the total protein levels of LATS but could significantly increase the phosphorylation of LATS. This suggests that CXCR7 may directly impact the phosphorylation status of LATS, while not affecting the phosphorylation of MST (Supplementary Fig. 7B). We carried out further experiments to address the role if B-arrestin is involved in the regulation between CXCR7 and YAP signaling. Interestingly, we observed a novel mechanism, which could explain the regulation of CXCR7 in YAP function. The immuno-precipitation data showed that beta-arrestin could associate with both yap and CXCR7 (Supplementary Fig. 7C). However, the activation of CXCR7 could enhance the interaction between CXCR7 and beta-arrestin but dissociate the complex between beta-arrestin and yap (Supplementary Fig. 7D), which lead to increased yap nuclear trans-location and target gene expression (Supplementary Fig. 7E; Fig. 5K and L). Thus, the regulation of CXCR7 on yap function could be due to two possible molecular mechanisms.

### YAP transcriptionally regulates CXCR7 expression, which forms a forward regulatory loop between Hippo/YAP and CXCR7

**Fig. 8:**
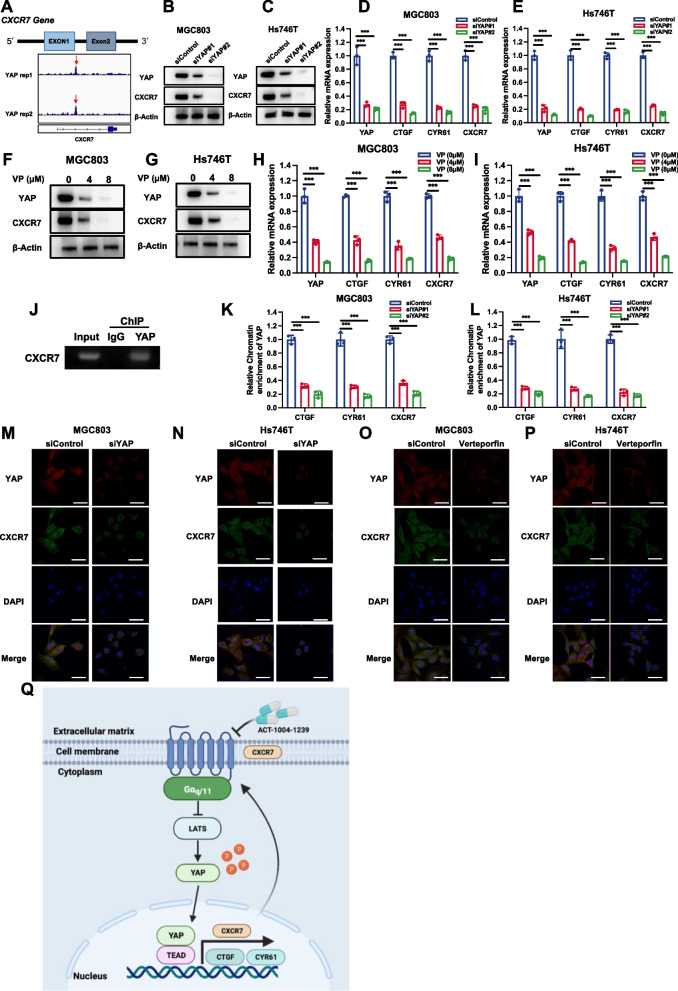
YAP transcriptionally regulates CXCR7 expression, which forms a forward regulatory loop between Hippo/YAP and CXCR7. **A** CXCR7 genome schematic and database analysis of the binding region of YAP to the CXCR7 promoter. **B**, **C** YAP depletion decreased CXCR7 protein levels in gastric cancer cells. MGC803 and Hs746T cells were transfected with YAP siRNA. Immunoblotting was performed with the indicated antibodies. **D**, **E** YAP depletion in MGC803 and Hs746T cells inhibited CXCR7 mRNA. MGC803 and Hs746T cells were transfected with siControl or siYAP. After 48 h, total RNA was extracted for gene expression analysis. Each group was tested in triplicate. *P < 0.05, **P < 0.01, ***P < 0.001 for comparisons of target gene expression. **F**, **G** VP treatment in MGC803 and Hs746T cells inhibited CXCR7 protein expression. MGC803 and Hs746T cells were treated with vehicle and VP. Immunoblotting was performed with the indicated antibodies. **H**, **I** VP treatment in MGC803 and Hs746T cells inhibited CXCR7 mRNA. MGC803 and Hs746T cells were treated with vehicle or VP. After 48 h, total RNA was extracted for gene expression analysis. Each group was tested in triplicate. *P < 0.05, **P < 0.01, ***P < 0.001 for comparisons of target gene expression. **J** ChIP assays showed that YAP could bind to the promoter region of CXCR7. MGC803 cells were fixed for 30 min. Rabbit IgG was used as the negative control. The primer sequences are shown in the Methods section. The enriched DNA fragments were subjected to PCR and DNA gel electrophoresis. **K**, **L** YAP silencing decreased binding to the promoter region of the CXCR7 gene. YAP was depleted in MGC803 and Hs746T cells, and ChIP-qPCR assays showed that YAP reduced binding to classical Hippo target genes, such as CTGF and CYR61, and reduced its binding to the CXCR gene. **M**-**P** YAP silencing or inhibition decreased CXCR7 expression in the membrane. MGC803 and Hs746T cells were transiently transfected with control or YAP siRNAs or were treated with VP. Endogenous YAP (green) and nuclei (blue) were stained with specific antibodies and DAPI, respectively; scale bar, 20 mm. Quantifications of YAP subcellular localization from at least 100 randomly selected cells. C, cytoplasm; N, nucleus. **Q** CXCR7 formed a regulatory loop with the Hippo/YAP axis in gastric cancer. The activation of CXCR7 could facilitate the Hippo/YAP axis and gastric tumour progression via the Gaq/11-ROCK-LATS axis. In turn, YAP could bind to the promoter region of the CXCR7 gene to promote its transcription

Since YAP is an important effector in modulating Hippo target gene expression, several studies have investigated the global genomic binding of YAP in several cancer cells [47]. By further analysis of YAP-based ChIP sequencing data, we found an obvious binding peak in the promoter region of CXCR7, which indicated the possible regulation of CXCR7 expression by YAP (Fig. 8A). We further depleted YAP in gastric cancer cells, which showed that YAP silencing decreased CXCR7 protein levels and mRNA levels in gastric cancer cells (Fig. 8B-E). To confirm this observation, we utilized VP (verteporfin) to block the interaction between YAP and TEADs. Consistently, VP treatment in gastric cancer cells decreased CXCR7 protein levels and mRNA levels (Fig. 8F-I). We further validated the interaction between the YAP protein and the promoter region of the CXCR7 gene by ChIP assays (Fig. 8J). ChIP assays showed that CTGF and CYR61 could bind to the promoter region of CXCR7 (Supplementary Fig. 8A and B). To validate this YAP-driven transcriptional activation, we depleted YAP in MGC803 and Hs746T cells, while the ChIP-qPCR assay showed that YAP depletion not only decreased YAP binding to classical Hippo target genes, such as CTGF and CYR61 but also reduced its binding to the CXCR gene (Fig. 8K, L). Finally, we carried out immunostaining after YAP depletion or VP treatment in gastric cancer cells. The immunostaining data showed that both YAP silencing and inhibition decreased CXCR7 expression in the membrane (Fig. 8M-P). These data indicated the direct regulation of YAP on CXCR7 expression in gastric cancer cells.

## Discussion

In the current study, we identified one GPCR family member, CXCR7, that forms a regulatory loop with the Hippo/YAP axis in gastric cancer. Clinically, CXCR7 expression correlated with the gene signature of the Hippo pathway and poor survival in gastric cancer. The activation of CXCR7 facilitates gastric tumour progression by modulating YAP phosphorylation and nuclear localization, while pharmaceutically targeting CXCR7 via ACT could promote YAP phosphorylation and cytosol retention, which subsequently inhibit gastric tumour growth. In addition, YAP could directly induce the expression of CXCR7, identifying a positive feedback loop between CXCR7 and Hippo signalling (Fig. 8Q).

CXCR7 was first cloned from a dog thyroid cDNA library and named receptor dog cDNA1 (RDC-1) [48]. Further studies showed that CXCR7 was the receptor for CXCL12. The activation of CXCR7 facilitated several downstream signalling pathways, including the GPCR-related kinase pathway and the AKT pathway. In addition, CXCR7 can form homodimers or heterodimers with CXCR4 to facilitate calcium mobilization and ERK phosphorylation [49–51]. The expression of CXCR7 is elevated in several human diseases, including multiple sclerosis, Alzheimer’s disease and malignancies [35, 52, 53]. Several studies revealed that the expression of CXCR7 correlated with the malignancy of tumours. For example, CXCR7 correlated with poor overall survival and risk of distant metastasis in liver cancer. A few mouse model-based studies indicated that targeting CXCR7 via nanobodies could inhibit tumour growth in head and neck carcinoma [54, 55]. Based on the importance of CXCR7, several studies attempted to uncover the potential mechanisms. For example, CXCR7 could facilitate head and neck tumour growth via TGF/Smad signalling. However, the function of CXCR7 is still largely unclear, especially in gastrointestinal malignancies. Our current study revealed the novel regulation of CXCR7 in modulating the Hippo pathway in gastric cancer. We report that an important regulatory component, CXCR7, is both upstream and downstream of the Hippo pathway, which provides novel knowledge on the crosstalk between CXCR signalling and the Hippo pathway.

The biological link between chronic inflammation and gastric cancer has been known for decades, and CXCR7 belongs to the network of inflammatory receptors. Previous studies showed that the expression of CXCR7 was also induced by several inflammatory factors, such as IL8 and IL6 [56, 57]. Our study revealed the oncogenic function of CXCR7 in gastric cancer, which might indicate the mechanism by which chronic inflammation facilitates tumour growth, possibly via CXCR7 signalling. However, the overactivation of the YAP axis was regarded as an important driver of the tumorigenesis of gastric cancer, while our study demonstrated that the expression of CXCR7 was also induced by the Hippo/YAP axis and facilitated gastric cancer progression via the Hippo pathway. Based on the knowledge above, we propose that CXCR7 might play a pivotal role in coordinating Hippo signalling and the inflammatory pathway to facilitate carcinogenic processes in gastric cancer.

Previous studies demonstrated that blocking the YAP/TEAD interaction was a promising strategy for Hippo-driven cancers [58, 59]. However, pharmaceutical drugs, such as verteporfin and Super-TDU [17, 22, 27], were proposed to block the YAP axis and failed to translate into the clinic in several preclinical studies in Hippo-driven cancer. Although several possible reasons could account for the failure of verteporfin and Super-TDU, one important issue is cell membrane penetration, since the inhibitors have to cross the cell membrane to block cytosolic protein interactions [60, 61]. However, membrane proteins account for 60% of the newly developed clinical drug targets, which are hotspots for pharmaceutical interest. Due to the high success rate of transmembrane proteins, we shifted our strategy to inhibited the upstream membrane receptors of the Hippo/YAP axis in gastric cancer. Based on the importance of CXCR7 in gastric cancer, we believe that blockade of CXCR7 could be a plausible strategy for gastric cancer treatment.

Interestingly, in the Cell Report study, Chen et al. identified CXCR7 expression could be inhibited by JQ1 inhibition, while Chip-seq data revealed the YAP could bind to CXCR7 promoter regions [62]. Besides, in the Cell & Bioscience paper, the authors showed that the activation of CXCR7 could facilitate YAP nuclear localization [63]. Our conclusion was supported from independent studies. However, they did not further investigate the function of YAP signaling, YAP phosphorylation or regulation loops. We further identified that expression correlation between Yap activation and CXCR7 level. Thus, our experimental data were even more detail and more mechanistic, which provide direct regulatory evidence for YAP in CXCR7 expression in gastric cancer.

## Conclusion

Our study uncovered interesting positive feedback between Hippo signalling and CXCR7 in promoting gastric cancer progression. Blockade of CXCR7, which stops the positive feedback loop, could be a promising strategy for gastric cancer therapeutics. Since blocking the YAP/TEAD interaction could be difficult through pharmaceutics, targeting upstream GPCRs could be a plausible way to modulate the Hippo pathway in gastric cancer.

### Supplementary Information

**Additional file 1:** **Supplementary Fig. 1.** A: ELISA experiment showing the concentration of CXCL12 in serum was approximately 3000 ng/mL, while the concentration of CXCL12 in the DMEM was too low to be detected. B: The database analysis showed 90% of gastric cell line does not expression CXCR4 (https://www.proteinatlas.org/). C: Western blot analyses showed CXCR4 was barely expressed in several gastric cancer cells. **Supplementary Fig. 2.** A: Western blot experiments showed the knockout efficiency of CXCR7. B-C: ELDA experiments showed a reduction of clonogeneic capacity in siCXCR7 groups in MGC803 and Hs746T cells. **Supplementary Fig. 3.** A: Western blot experiments show that pYAP expression increases with increasing ACT concentration. B-C: ELDA experiments showed CXCR7 inhibitor ACT could significantly decrease the capacity of clonogenesis in MGC803 and Hs746T cells. **Supplementary Fig. 4.** A: Western blot experiments show that pYAP expression decreases with increasing ACT concentration. B-C: ELDA experiments showed CXCR7 activator ACT could significantly increase the capacity of clonogenesis in MGC803 and Hs746T cells. **Supplementary Fig. 5.** A: Western blot experiments showed the knockout efficiency of CXCR7. B: Western blot experiments showed the effects of ACT and TC on YAP phosphorylation. C-E: Western blot experiments showed the effects of ACT and TC on TAZ phosphorylation. **Supplementary Fig. 6.** A-B: ELDA experiments showed CXCR7 deletion reduced the clonogenic number of gastric cancer cells, which was further augmented by YAP overexpression. **Supplementary Fig. 7.** A: WB experiments showed that Gαq/11 activation can reduce the yap phosphorylation changes altered by CXCR7 deletion, but Gαs does not have this effect. B: Western blot experiments showed the effects of knocking out CXCR7 on MST and LATS. C: The immuno-precipitation data showed that beta-arrestin could associate with both yap and CXCR7. D: The activation of CXCR7 could enhance the interaction between CXCR7 and beta-arrestin but dissociate the complex between beta-arrestin and yap. E: The activation of CXCR7 could lead to increased yap nuclear trans-location. **Supplementary Fig. 8.** A-B: ChIP assays showed that CTGF and CYR61 could bind to the promoter region of CXCR7.

## Data Availability

RNA sequence data can be found in the GEO database (GSE233094). The original digital data and WB data are provided in the supplementary materials.

## Abbreviations

GPCR: G-coupled protein receptor
CXCR7: C-X-C chemokine receptor 7
ACT: ACT-1004–1239
TC: TC14012
ChIP: Chromatin immunoprecipitation
GSEA: Gene signature enrichment analysis
YAP: Yes-associated protein
TEAD: TEA domain transcription factor
LATS: Large tumour suppressor kinase
VP: Verteporfin
ROCK: Rho-associated protein kinase
