# Supplementary material for: Regulation of the Hippo/YAP axis by CXCR7 in the tumorigenesis of gastric cancer
Source: J Exp Clin Cancer Res. 2023 Nov 10;42:297. doi: 10.1186/s13046-023-02870-3 (PMC10636825; doi:10.1186/s13046-023-02870-3)

Supplementary Figure1

A

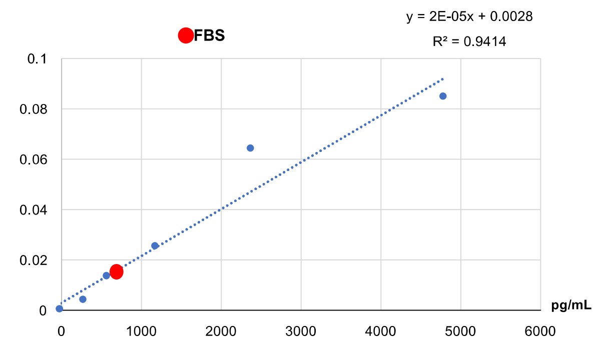

B

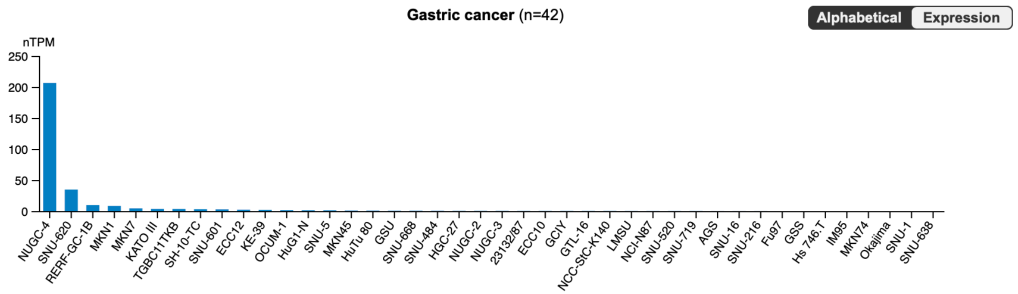

C

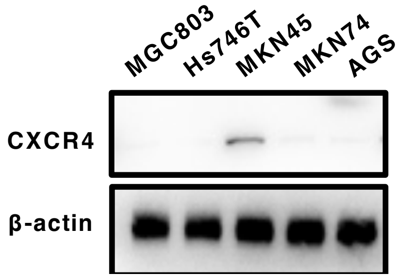

Supplementary Figure2

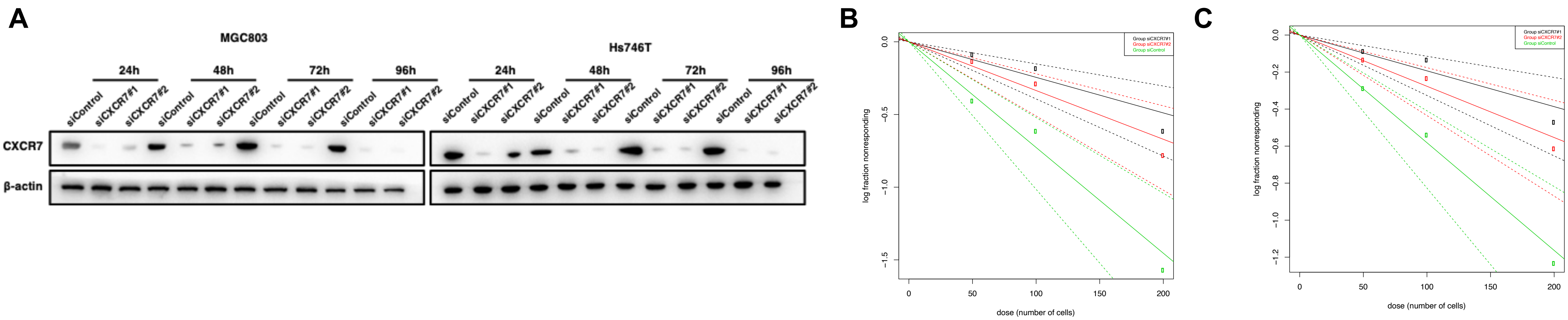

Supplementary Figure3

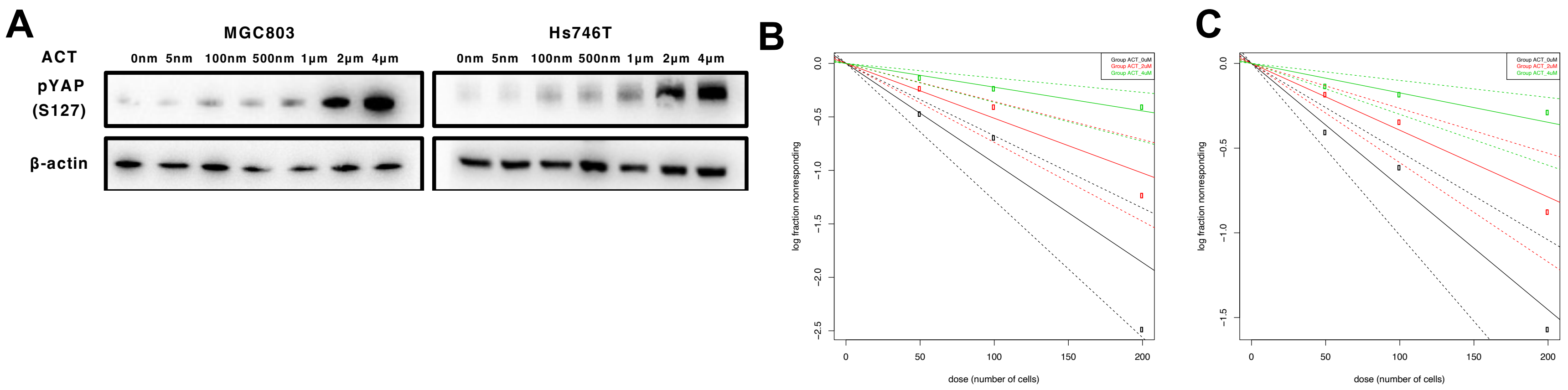

Supplementary Figure4

A

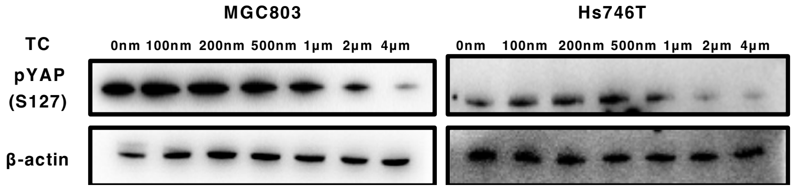

B

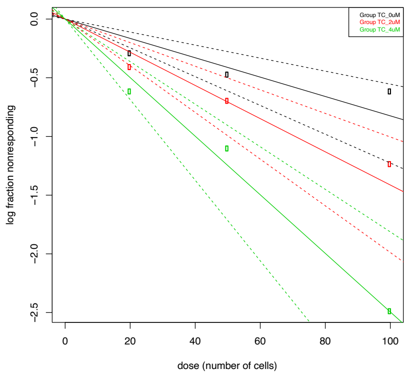

C

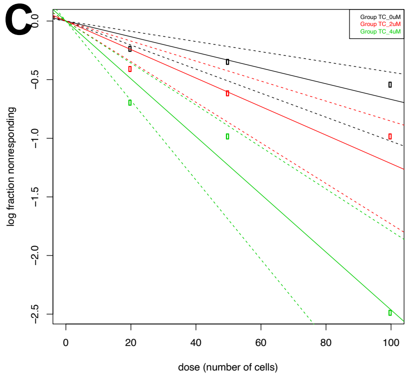

Supplementary Figure5

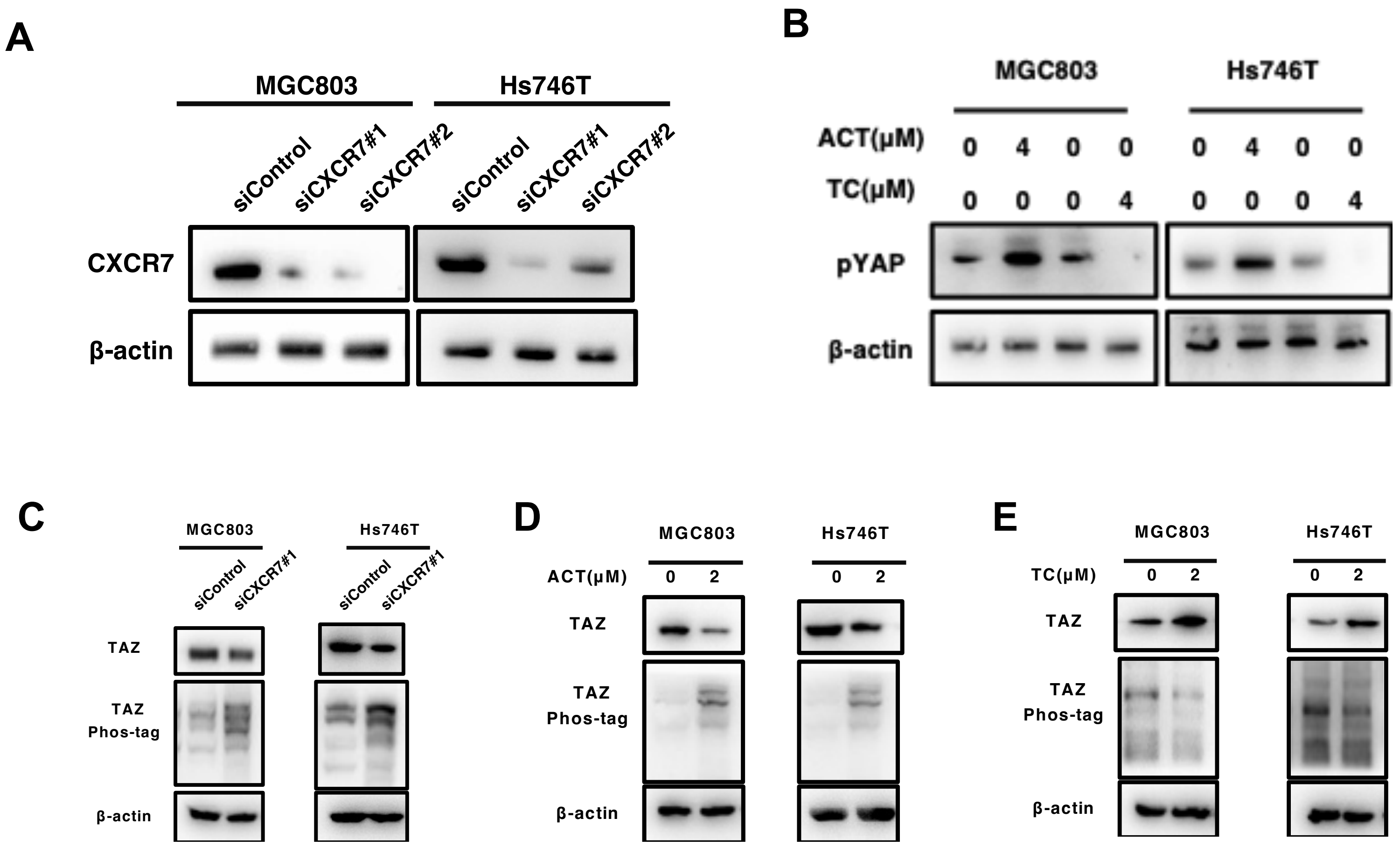

Supplementary Figure6

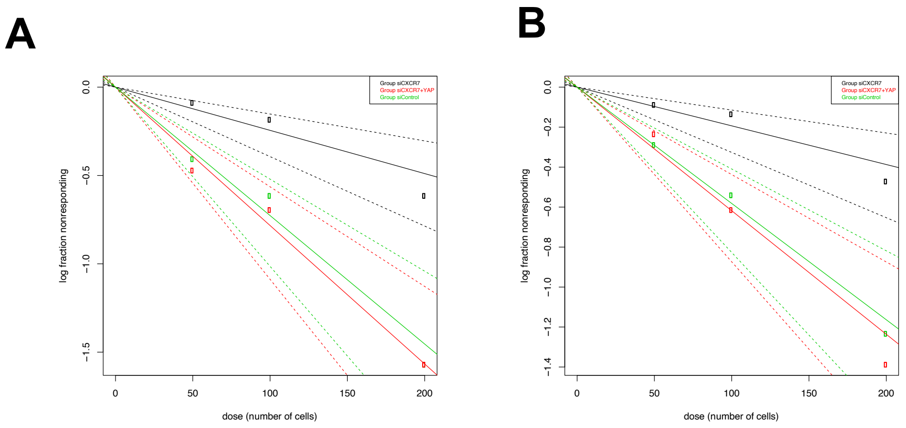

Supplementary Figure7

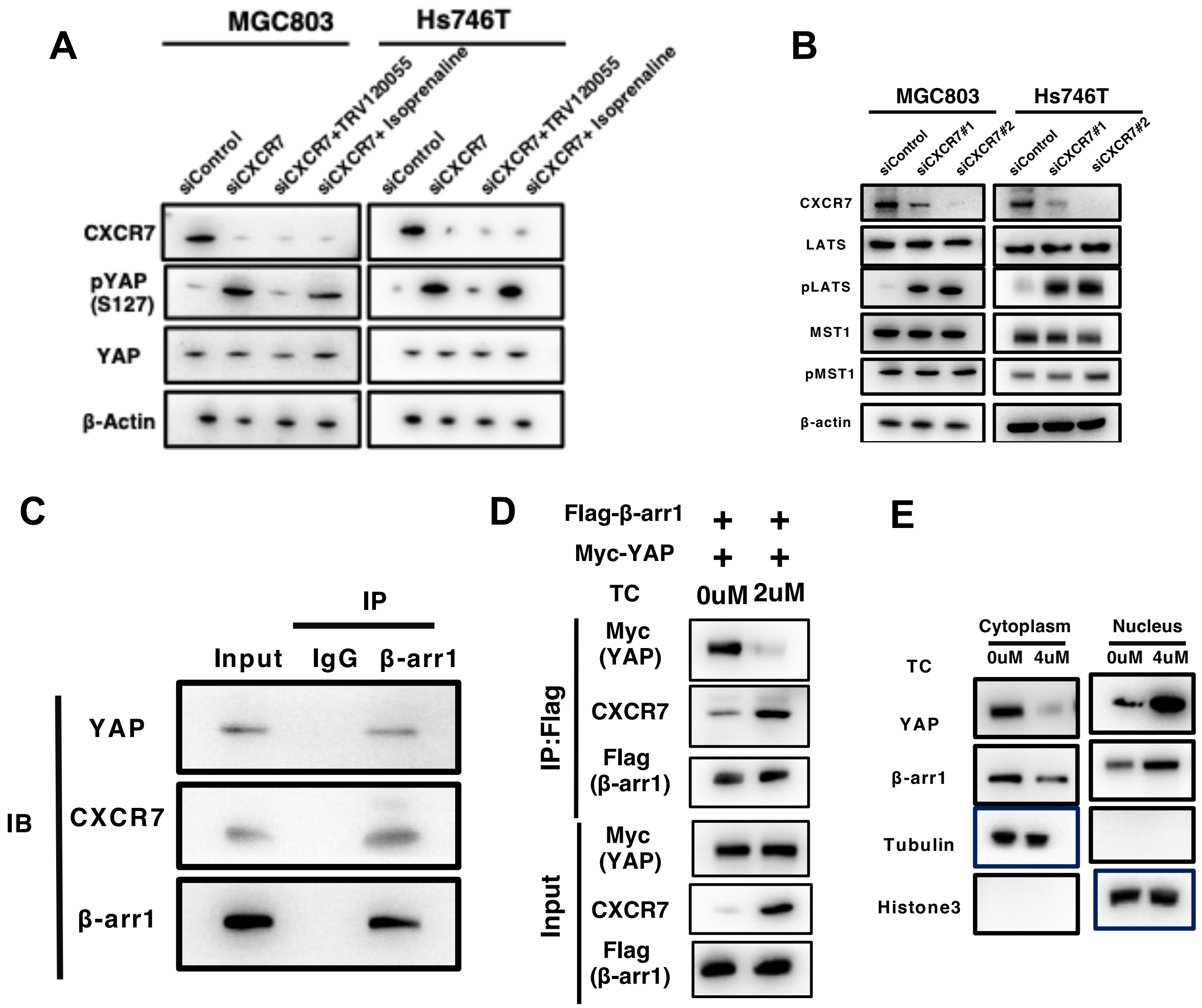

Supplementary Figure8

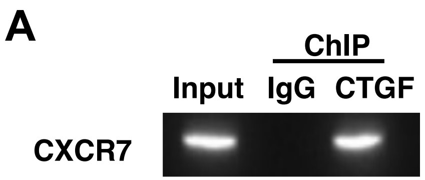

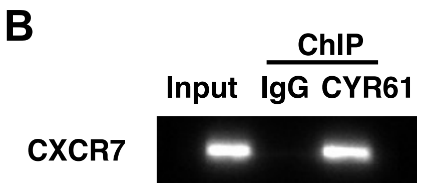

Supplement: Supplementary file 1 — Additional file 1: Supplementary Fig. 1. A: ELISA experiment showing the concentration of CXCL12 in serum was approximately 3000 ng/mL, while the concentration of CXCL12 in the DMEM was too low to be detected. B: The database analysis showed 90% of gastric cell line does not expression CXCR4 (https://www.proteinatlas.org/). C: Western blot analyses showed CXCR4 was barely expressed in several gastric cancer cells. Supplementary Fig. 2. A: Western blot experiments showed the knockout efficiency of CXCR7. B-C: ELDA experiments showed a reduction of clonogeneic capacity in siCXCR7 groups in MGC803 and Hs746T cells. Supplementary Fig. 3. A: Western blot experiments show that pYAP expression increases with increasing ACT concentration. B-C: ELDA experiments showed CXCR7 inhibitor ACT could significantly decrease the capacity of clonogenesis in MGC803 and Hs746T cells. Supplementary Fig. 4. A: Western blot experiments show that pYAP expression decreases with increasing ACT concentration. B-C: ELDA experiments showed CXCR7 activator ACT could significantly increase the capacity of clonogenesis in MGC803 and Hs746T cells. Supplementary Fig. 5. A: Western blot experiments showed the knockout efficiency of CXCR7. B: Western blot experiments showed the effects of ACT and TC on YAP phosphorylation. C-E: Western blot experiments showed the effects of ACT and TC on TAZ phosphorylation. Supplementary Fig. 6. A-B: ELDA experiments showed CXCR7 deletion reduced the clonogenic number of gastric cancer cells, which was further augmented by YAP overexpression. Supplementary Fig. 7. A: WB experiments showed that Gαq/11 activation can reduce the yap phosphorylation changes altered by CXCR7 deletion, but Gαs does not have this effect. B: Western blot experiments showed the effects of knocking out CXCR7 on MST and LATS. C: The immuno-precipitation data showed that beta-arrestin could associate with both yap and CXCR7. D: The activation of CXCR7 could enhance the interaction between CXCR7 a [file 13046_2023_2870_MOESM1_ESM.pdf]
